# Systematic identification of NF90 target RNAs by iCLIP analysis

**DOI:** 10.1038/s41598-021-04101-1

**Published:** 2022-01-10

**Authors:** Valeria Lodde, Matteo Floris, Rachel Munk, Jennifer L. Martindale, Davide Piredda, Catello Mario Panu Napodano, Francesco Cucca, Sergio Uzzau, Kotb Abdelmohsen, Myriam Gorospe, Ji Heon Noh, M. Laura Idda

**Affiliations:** 1 Department of Biomedical Sciences, University of Sassari, Sassari, Italy; 2 Laboratory of Genetics and Genomics, National Institute on Aging Intramural Research Program, National Institutes of Health, Baltimore, MD 21224 USA; 3 Department of Biochemistry, Chungnam National University, Daejeon, Korea; 4 Institute for Genetic and Biomedical Research (IRGB-CNR), Sassari, Italy; 5 Intensive Care Unit, Emergency Department, AOU Sassari, Sassari, Italy; 6 Internal Medicine Unit, ATS Sardegna, Ospedale Civile Alghero, Sassari, Italy; 7 Microbiology and Virology Unit, Diagnostic Department, AOU Sassari, Sassari, Italy

**Keywords:** Cell biology, Immunology, Molecular biology, Biomarkers, Molecular medicine

## Abstract

RNA-binding proteins (RBPs) interact with and determine the fate of many cellular RNAs directing numerous essential roles in cellular physiology. Nuclear Factor 90 (NF90) is an RBP encoded by the interleukin enhancer-binding factor 3 (*ILF3*) gene that has been found to influence RNA metabolism at several levels, including pre-RNA splicing, mRNA turnover, and translation. To systematically identify the RNAs that interact with NF90, we carried out iCLIP (individual-nucleotide resolution UV crosslinking and immunoprecipitation) analysis in the human embryonic fibroblast cell line HEK-293. Interestingly, many of the identified RNAs encoded proteins involved in the response to viral infection and RNA metabolism. We validated a subset of targets and investigated the impact of NF90 on their expression levels. Two of the top targets, *IRF3* and *IRF9* mRNAs, encode the proteins IRF3 and IRF9, crucial regulators of the interferon pathway involved in the SARS-CoV-2 immune response. Our results support a role for NF90 in modulating key genes implicated in the immune response and offer insight into the immunological response to the SARS-CoV-2 infection.

## Introduction

RNA-binding proteins (RBPs) robustly influence gene expression programs by modulating a range of post-transcriptional steps, including pre-mRNA splicing and processing, as well as mRNA export to the cytoplasm, mRNA turnover, and translation^1^. Consequently, RBPs are major factors in the response of cells to both intracellular and extracellular stimuli. Nuclear Factor 90 (NF90) is a multifunctional DNA- and RNA-binding protein found mainly in the nucleus and encoded by the interleukin enhancer-binding factor 3 (*ILF3*) gene. The role of NF90 as a DNA-binding transcription factor was first identified in 1994 by Crothesy and colleagues while studying the human transcription factor complex NFAT (nuclear factor of activated T cells), which is involved in the transcriptional regulation of the *IL2* and *IL13* genes^2,3^. Deeper characterization of NF90 as transcription factor revealed that it induces the proliferation of K562 erythroleukemia cells and regulates the expression of immediate early genes^4,5^, genes which are rapidly activated in response to several stimuli^6,7^.

As an RBP, NF90 possesses two double-stranded RNA-binding motifs (dsRBM), a zinc finger domain (ZNF), and a nucleic acid-binding arginine/glycine-rich (RGG) motif^8^, through which it binds single- and double-stranded RNA from viruses and mammalian cells^4^. In turn, NF90 modulates RNA metabolism by influencing mRNA splicing, export to the cytoplasm, turnover, and translation^5,9–11^. NF90 regulates mRNA stability and translation efficiency by binding to the 3'-untranslated region (UTR) of target mRNAs, many of them encoding proteins involved in cell proliferation and in the immune response. NF90 was shown to bind a subset of RNAs containing AU-rich sequences in the 3'UTR, including *IL2* mRNA during T cell activation and *MKP-1* mRNA in response to oxidative damage^9,12^. NF90 stabilizes the mRNAs encoding the cyclin-dependent kinase inhibitor CDKN1A (p21) and the myogenic transcription factor MYOD in developing skeletal muscle^13^, while it promotes cell proliferation by binding the 3'UTR of *CCNE1* mRNA, encoding cyclin E1, and stabilizing it, an effect that was further enhanced by the long noncoding (lnc) RNA *LINC00470*^14,15^. NF90 was found to suppress the translation of several mRNAs encoding proinflammatory factors; NF90 levels declined in senescent fibroblasts, leading to derepression and increased translation of several inflammatory cytokines^16^. Our studies found that NF90 regulates the stability of several cytokines following exposure to *Plasmodium falciparum* antigens^17^, repressed the translation of the cytokine BAFF in THP-1 cells, and cooperated with microRNA miR-15a in binding *BAFF* mRNA to inhibit translation^18,19^.

In order to systematically identify the collection of NF90 target mRNAs and further determine the sites of interaction with target mRNAs, we carried out iCLIP (individual nucleotide resolution UV cross-linking and immunoprecipitation) analysis in HEK-293 cells. We found several RNAs that encoded proteins implicated in RNA metabolism, the response to viral infection, and signaling through RAP1. We validated these interactions by ribonucleoprotein (RNP) immunoprecipitation (RIP) analysis and investigated the impact of NF90 on the stability and translation of these target mRNAs. Among the most interesting NF90 targets, we identified several mRNAs encoding transcription factors in the interferon regulatory factor (IRF) family^20^ and YWHAH, a protein implicated in susceptibility for schizophrenia and Parkinson’s disease^21,22^, as well as the lncRNA *XIST*, a known NF90 target^23^. IRFs are implicated in processes including the regulation of innate immunity and cell cycle progression^20^, and were recently shown to have a relevant role in the immune response during infection by the coronavirus SARS-CoV-2^24–26^. Our results support a role of NF90 as a major regulator of RNA metabolism in a range of cellular responses, particularly viral infection.

## Results

### Identification of NF90 target mRNAs

**Figure 1:**
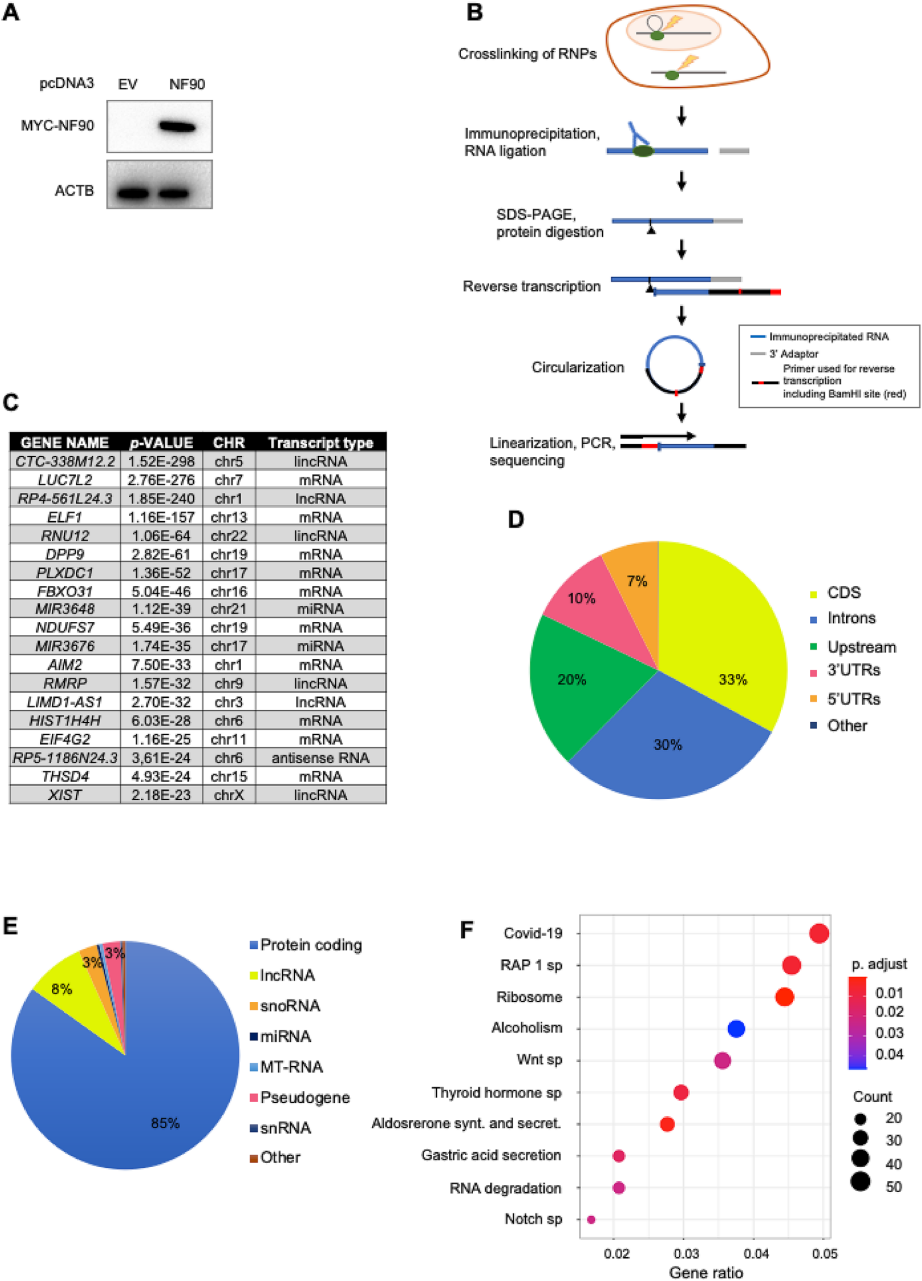
Identification of NF90 target mRNAs in HEK-293 cells by iCLIP analysis. **(A)** Western blot analysis of MYC-NF90 in HEK-293 cells overexpressing a wild-type, full-length, MYC-tagged human NF90. The levels of ACTB (β-Actin) were assessed to monitor even loading of samples. **(B)** Schematic representation of the iCLIP methodology. Cells transfected with MYC-NF90 were initially crosslinked with 254 nm UV before being lysed. The MYC-NF90 RNP complexes were immunoprecipitated and visualized after ^32^P-end-labeling of the RNA, followed by size-separation through SDS-containing polyacrylamide gels and transfer to nitrocellulose membranes. After the MYC-NF90 complexes were cut from the membrane and digested with Proteinase K, the RNA was recovered from the membrane and reverse transcribed into cDNA. After circularization, followed by linearization and PCR amplification, the RNA fragments were identified by RNA-sequencing. **(C)** Most abundant NF90 RNA targets identified by iCLIP analysis; *p*-value of the association, chromosome (Chr) from where the RNA is transcribed and type of RNA are indicated. **(D)** Percentage of all NF90 iCLIP reads mapping to RNA subdivided by transcript type/region. **(E)** Percentage of RNA type identified by our analysis. **(F)** KEGG pathway enrichment analysis of the NF90-target mRNAs identified by iCLIP.

The collective of NF90 target RNAs in human HEK-293 cells was examined by overexpressing MYC-tagged NF90 (Fig. 1A) to isolate NF90 ribonucleoprotein (RNP) complexes. After crosslinking using ultraviolet light, a MYC-specific antibody was used to immunoprecipitate NF90 RNPs; the complexes were end-labeled using ^32^P-ATP, size-separated by using SDS-containing polyacrylamide gels, transferred to a nitrocellulose membrane, and recovered from the membrane by digesting the protein with proteinase K. After reverse transcription of the RNA into cDNA, circularization, and linearization, the protected RNA was identified by high-throughput RNA-sequencing (RNA-seq) analysis (Fig. 1B).

The analysis revealed 2936 NF90 target transcripts; a partial list of NF90 target is presented in Fig. 1C, and a complete list in Supplementary Table S1. iCLIP signals were preferentially found within CDS (coding DNA sequence) regions and untranslated regions (UTRs) (50% in total) of protein-coding mRNAs (85%) (Fig. 1D,E). Analysis of the distribution of the signals throughout RNAs expressed from the different chromosomes (Chr.) revealed that most of the signals are present at level of the Chr. 1 and 19, suggesting that there is no apparent correlation between chromosome length and number of signals (R^2^ = 0.02036, p-value = 0.516). Coverage profile of query regions at feature boundaries indicated that the signals distributed preferentially towards the end of the 5'UTR and the beginning of the 3'UTR (Supplemental Fig. S1A,B). Transcripts interacting specifically with NF90 were used to computationally identify the presence of conserved RNA signature motifs. Among the candidate motifs identified from our dataset, five motifs showed the highest frequency of hits (Supplemental Fig. S1C). In line with previous results^27^ these motifs were highly enriched in A and U residues.

We next performed Reactome pathway (Reactome PA), Kyoto Encyclopedia of Genes and Genome (KEGG) pathway analyses and Gene Ontology (GO) to identify functional information from NF90 targets. Reactome PA revealed that the proteins encoded by NF90 target mRNAs were mainly implicated in biological processes related to RNA degradation, SARS-CoV-2 infection, and WNT signaling pathways. Similarly, enriched KEGG pathways included eukaryotic translation, nonsense-mediated RNA decay independent of exon junction complexes, and regulation of viral translation (Fig. 1F, Supplemental Fig. S1D). These data indicate that NF90 target RNAs are involved in important cellular processes including metabolism of mammalian and viral RNA. GO analysis of the iCLIP targets identified an enrichment in genes implicated in cell–cell interactions and in particular in neuronal interactions, as well as many ribosomal RNA-related functions (Cellular Component, Supplemental Fig. S2A). Regarding biological processes, we found several target RNAs implicated in RNA and viral metabolism, and several genes implicated in neural and epithelial tube formation pathways (Supplemental Fig. S2B).

### Validation of endogenous NF90 target transcripts

**Figure 2:**
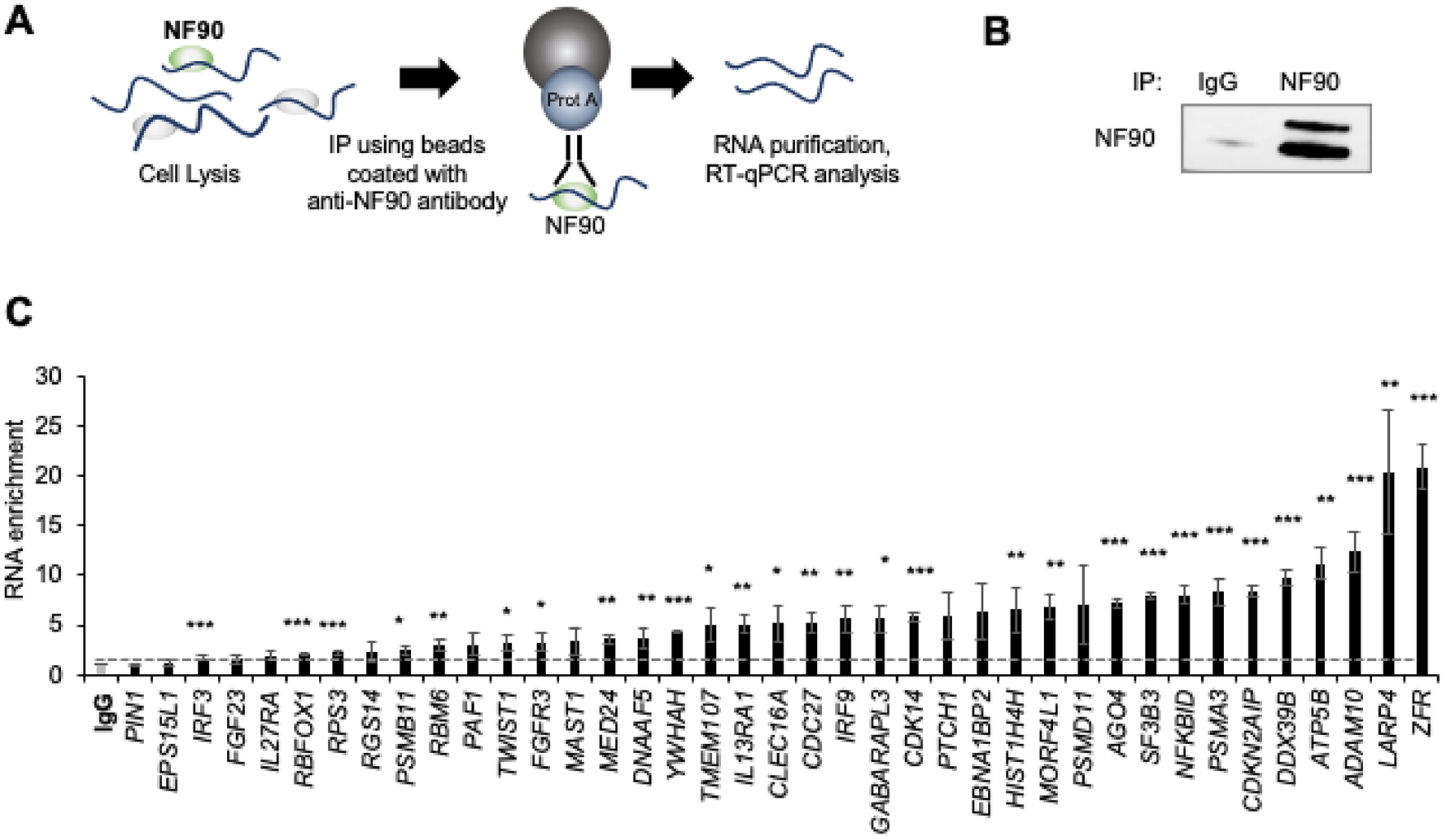
Validation of the mRNA targets using Ribonucleoprotein immunoprecipitation. **(A)** Schematic representation of the Ribonucleoprotein immunoprecipitation (RIP) analysis. **(B)** After immunoprecipitation (IP) using anti-NF90 or IgG antibodies, the presence of NF90 in the IP material was assayed by Western blot analysis. **(C)** RIP analysis was conducted using lysates from HEK-293 cells and anti-NF90 or IgG antibodies. The levels of target mRNAs in the ribonucleoprotein (RNP) complexes present in the IgG IP and the NF90 IP samples were analysed by RT-qPCR and the levels of enrichment of the mRNAs in NF90 IP relative to IgG IP were calculated after normalization to *ACTB* mRNA levels in each IP group. Data are the means and standard deviation (+ SD) from at least three independent experiments. *, P < 0.05; **, P < 0.01, ***, P < 0.005.

We validated a subset of NF90 target RNAs identified by iCLIP analysis by performing RNP IP (RIP) analysis using native (non-denaturing) conditions that preserved the binding of endogenous RNAs to NF90, and measuring their enrichment in NF90 RIP samples by reverse transcription (RT) followed by quantitative (q)PCR analysis^28^. NF90 RNP complexes were isolated from HEK-293 lysates using a specific antibody directed at NF90 (Fig. 2A); in parallel, control IP reactions were carried out using pre-immune IgG. Western blot analysis was used to detect NF90 in the anti-NF90 IP samples (Fig. 2B). Purified RNAs were then measured by RT–qPCR analysis employing specific primer pairs as previously described^28^. The vast majority of transcripts tested by RIP analysis confirmed the iCLIP data; some transcripts, such as *ZFR*, *LARP4*, *ADAM10* and *ATP5B* mRNAs, showed particularly high enrichment levels in NF90 IP as compared to IgG IP samples, as measured by RT–qPCR analysis (Fig. 2C). A small percentage of the targets identified with the iCLIP, such as *PIN1* and *EPS15L1* mRNAs, were not validated by RIP analysis; in general, these may represent target transcripts in which NF90 binds introns or mRNA isoforms that were not amplified by the primers selected. Binding of NF90 to *ACTB* mRNA, which encodes a housekeeping protein, was measured in order to normalize data input, since *ACTB* mRNA is not a target of NF90 and thus the *ACTB* mRNA detected represented the background RNA binding in a nonspecific manner to beads, plastic, and other components of the IP reactions. A small number of lncRNAs was also analyzed by RIP analysis (Supplemental Fig. S3A). In particular, we observed a strong interaction of NF90 with the lncRNA *XIST*, a key lncRNA involved in the regulation of X chromosome inactivation^29^. Interestingly, in cells expressing low levels of NF90, all the lncRNAs analyzed were downregulated (Supplemental Fig. S3B). Due to the relevance of *XIST* we decided to show a representative genome browser snapshots of the iCLIP peaks (Supplemental Fig. S3C).

**Figure 3:**
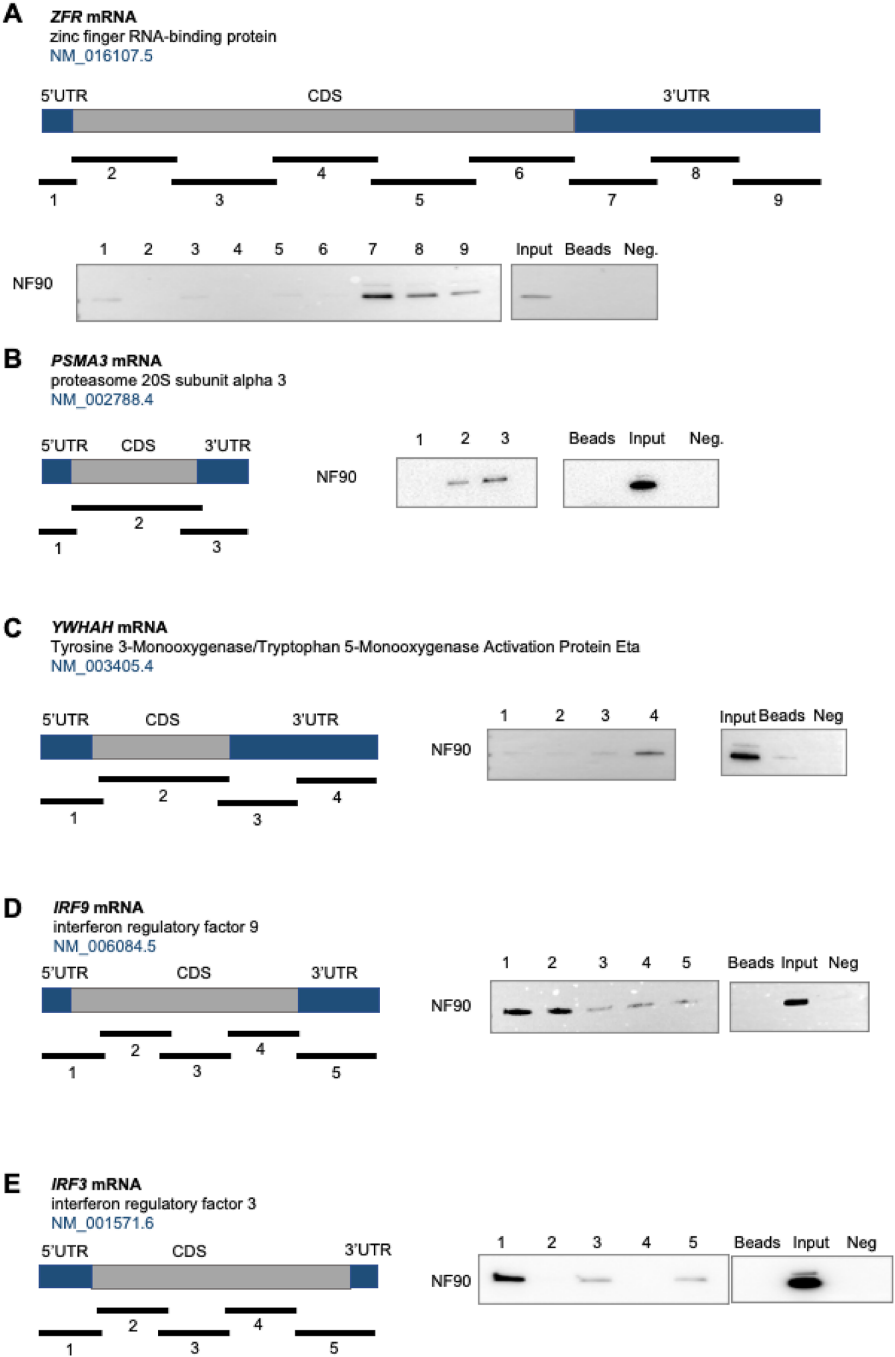
Biotin pulldown assays to assess NF90 binding to target mRNAs. The biotinylated RNAs (heavy black bars) synthesized for each mRNA were incubated with cytoplasmic lysates from HEK-293 cells; after pulldown of proteins associated with the biotinylated RNAs, the presence of NF90 was assessed by western blot analysis. Here, we specifically analyzed the interaction of NF90 with biotinylated RNAs spanning different regions of *ZFR* mRNA **(A)**, *PSMA3* mRNA **(B)**, *YWHAH* mRNA **(C)**, *IRF9* mRNA **(D)**, and *IRF3* mRNA **(E)**. *Beads* (no biotinylated RNA); *Input*, 20 μg lysate; *Neg*., biotinylated fragment of *BAFF* 3'UTR RNA. Immunoblotting image of Input, beads and negative control are from two different gels.

We further confirmed the interactions of NF90 with target RNAs using biochemical methods. Five NF90 target mRNAs were biotinylated using in vitro transcription methods, and their interactions with NF90 were monitored by biotin-pull down analysis. Specifically, we analyzed the binding of NF90 with partial biotinylated RNAs corresponding to the Zinc Finger RNA Binding Protein (*ZFR*) and proteasome 20S subunit alpha 3 (*PSMA3*) mRNAs, which showed strong interaction with NF90 by RIP analysis, the Tyrosine 3-Monooxygenase/Tryptophan 5-Monooxygenase Activation Protein Eta (*YWHAH*) mRNA, which showed weaker interaction, and two transcripts encoding Interferon Regulatory Factors (IRFs), which are associated with SARS-CoV-2 immune response, *IRF3* and *IRF9* mRNAs (Fig. 3A–E). Biotinylated transcripts spanning the 3’UTR, CDS and 5’UTR of these mRNAs were synthesized in vitro and subsequently incubated with HEK-293 cell lysates. The biotinylated RNA–protein complexes were pulled down using streptavidin-coated beads and the presence of NF90 in association with the biotinylated RNA was analyzed using western blot analysis. As shown (Fig. 3), binding was validated for all the biotinylated RNAs tested (*ZFR*, *PMSA3, YWHAH, IRF3* and *IRF9* mRNAs). *ZFR*, *PMSA3, YWHAH* mRNAs showed stronger binding at the 3’UTR RNAs, while for *IRF3* and *IRF9* mRNAs, preferential binding was seen with the 5’UTR RNAs. Biotinylated RNA fragments from coding regions of *PSMA3* and *IRF9* mRNAs were also found to bind NF90. A biotinylated fragment synthesized to contain a region of *BAFF* 3'UTR mRNA, which we previously demonstrated not to bind with NF90^30^, was included as a negative control (Neg) (Fig. 3A–E).

### NF90 regulates mRNAs and proteins involved in SARS-CoV-2 immune response

**Figure 4:**
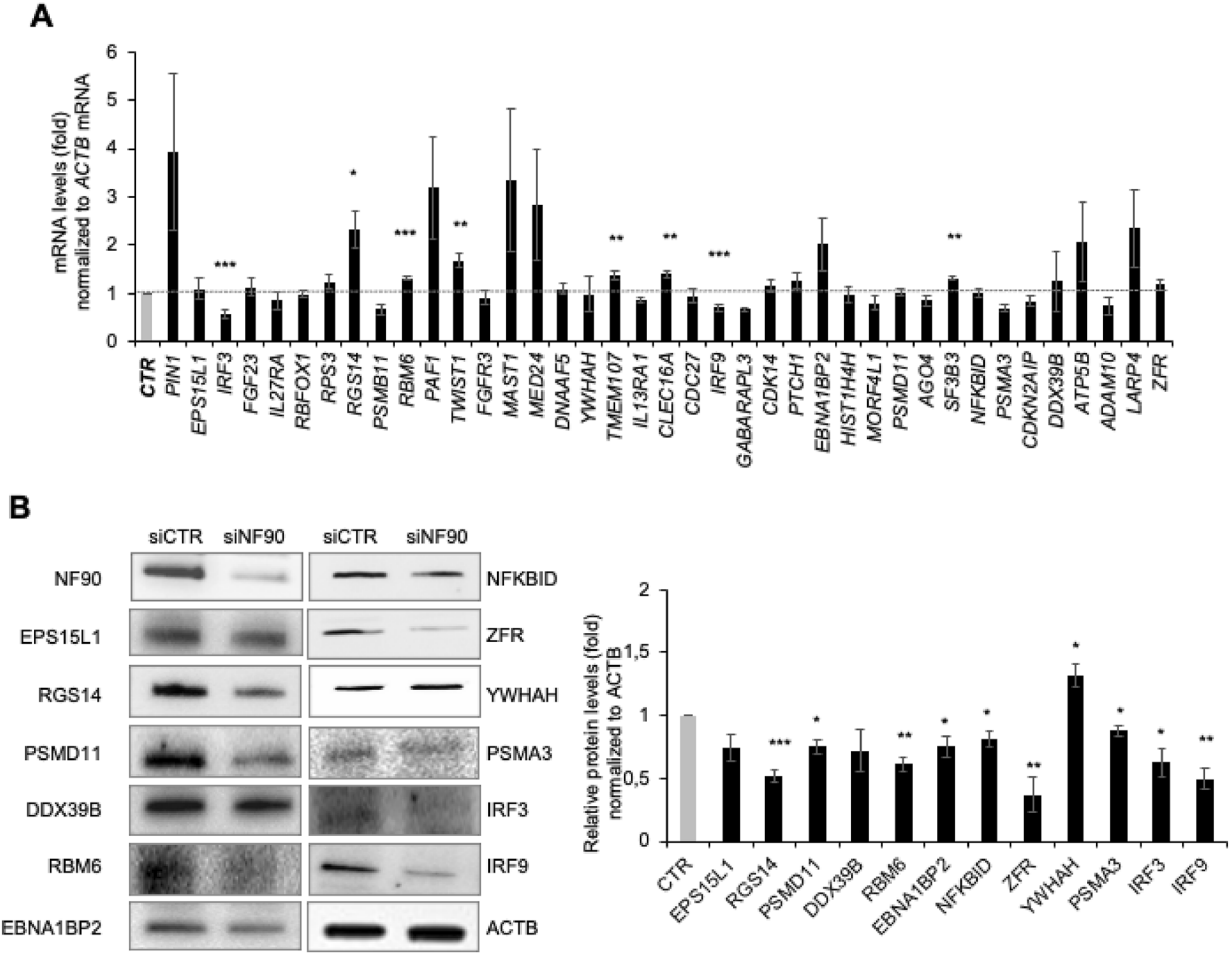
NF90 silencing modulates the level of select target mRNAs. HEK-293 cells were transfected to express normal (CTR siRNA) or reduced (NF90 siRNA) NF90 levels; 48 h after transfection, the abundance of selected mRNAs and proteins was measured by RT-qPCR analysis **(A)** and Western blot analysis **(B)**, respectively. In (**B**) NF90 and ACTB proteins were also assessed by Western blot analysis to monitor the efficiency of the silencing intervention and identify changes in loading between the samples, respectively. Quantification of the Western blot (right panel) were performed using ImageJ. Data in **(A)** are the means and standard deviation (+ SD) from at least three independent experiments. *, P < 0.05; **, P < 0.01, ***, P < 0.005. *CTRL,* siRNA control.

To investigate the influence of NF90 on the fate of target RNAs, we first assessed the steady-state levels of mature target mRNAs after NF90 silencing. NF90 was silenced using small interfering (si)RNA specifically targeting *ILF3* mRNA (which encodes NF90) and 48 h later, total RNA was collected, and the levels of target mRNAs were measured by RT-qPCR analysis. NF90 downregulation did not dramatically influence the steady-state levels of most mRNAs analyzed (Fig. 4A), despite robust silencing (Fig. 4B). The most significant effects were observed for *IRF3* and *IRF9* mRNAs. These mRNAs were strongly downregulated in NF90-silenced samples, while *RGS14*, *TWIST1* and *CLEC16A* mRNAs were modestly elevated.

The impact of NF90 on total RNA levels was tested globally by RNA sequencing (RNA-seq) analysis of HEK-293 cells in which NF90 was left unchanged or was silenced, as shown in Fig. 4 (Supplemental Fig. S4A). Comparison of total RNA levels in cells expressing low levels of NF90 (NF90 siRNA) to cells expressing normal levels of NF90 (Ctrl siRNA) identified 854 genes differentially expressed, 265 significantly upregulated and 589 significantly downregulated (Supplemental Fig. S4B; Supplementary Table S2 for complete list). For the RNAs most differentially abundant, relative (fold) changes were calculated by comparing RNA-seq results from NF90 siRNA cells with Ctrl siRNA (Supplemental Fig. S4C,D). In line with our iCLIP results, among the most differentially expressed mRNAs were those encoding eukaryotic and viral translation factors, as assessed by KEGG pathway analysis (Supplemental Fig. S4E). However, comparison of the mRNA targets identified by iCLIP and those found by RNA-seq analysis revealed only a small overlap of 3.7% (134 genes) between the two RNA sets, supporting a substantial role for NF90 in regulating translational events as an RNA-binding protein in HEK-293 cells (Supplemental Fig. S4F). Interestingly, among the 134 genes overlapping between the two analysis we observed a trend towards more downregulated genes, 99 (73%) out of 134. This trend suggests that NF90 positively regulates the levels of many target RNAs.

To study if the impact of NF90 on target mRNAs was associated with changes in the levels of encoded proteins, we performed Western blot analysis of several target mRNAs and quantified them using ImageJ (Fig. 4B, left and right panels). Interestingly, a greater magnitude of effect was seen at the protein level (Fig. 4B) than at the mRNA level (Fig. 4A). Furthermore, most proteins showed reduced abundance after NF90 silencing, with the exception of YWHAH, which appeared slightly elevated. Overall, these results support a model whereby in basal conditions, NF90 binds to many mRNAs in HEK-293 cells and does not substantially affect their abundance, and instead appears to lower the steady-state abundance of proteins encoded by the target mRNAs.

### NF90 silencing affects the stability and translation of some NF90 target mRNAs

**Figure 5:**
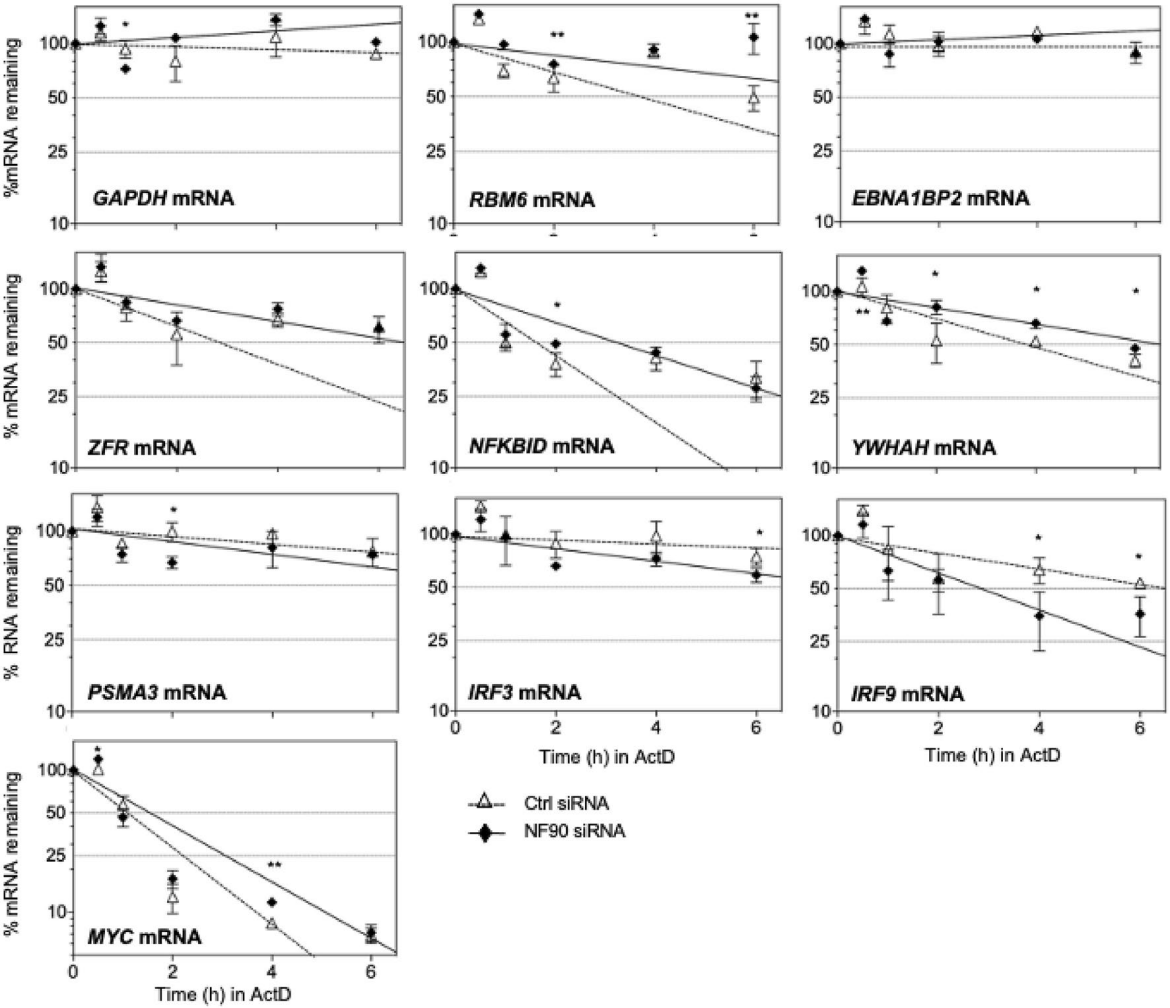
Influence of NF90 on the stability of target mRNAs. HEK-293 cells that were processed as described in Fig. 4 to express either normal (Ctrl siRNA) or reduced (NF90 siRNA) levels were used to assess the relative rates of clearance of NF90 target mRNAs by RT-qPCR analysis after treatment with actinomycin D for the indicated times. *GAPDH* mRNA, a stable transcript, and *MYC* mRNA, an unstable transcript, were included as controls. The levels of mRNAs were first normalized to *18S* mRNA levels and then plotted using GraphPad Prism on semi-logarithmic scales relative to 100%, the abundance of each mRNA before adding actinomycin D. Data are the means and standard deviation (+ SD) from at least three independent experiments. *, P < 0.05; **, P < 0.01, ***, P < 0.005.

The results showed in Fig. 4 demonstrated that the silencing of NF90 did not significantly modify the steady-state levels of the endogenous target mRNAs analyzed (Fig. 4A). To directly test if NF90 controls the stability of the selected mRNAs we measured the half-lives of a subset of NF90 target mRNAs by treating cells with actinomycin D (an inhibitor of RNA polymerase II, used here to block de novo transcription) and measuring the rate of mRNA decay. In cells transfected with Ctrl or NF90 siRNAs actinomycin D was added 24 h later, total RNA was collected at the times shown, and mRNA levels were measured to evaluate the rates of decline (Fig. 5). Although the rates of decline were not totally equal, and silencing NF90 seemed to render some mRNAs slightly more stable (Fig. 5), the differences were quite minor at steady-state conditions. The loss of unstable *MYC* mRNA was included in the analysis as a positive control for the effect of actinomycin D^31^, while a stable transcript, *GAPDH* mRNA, was used as a negative control.

**Figure 6:**
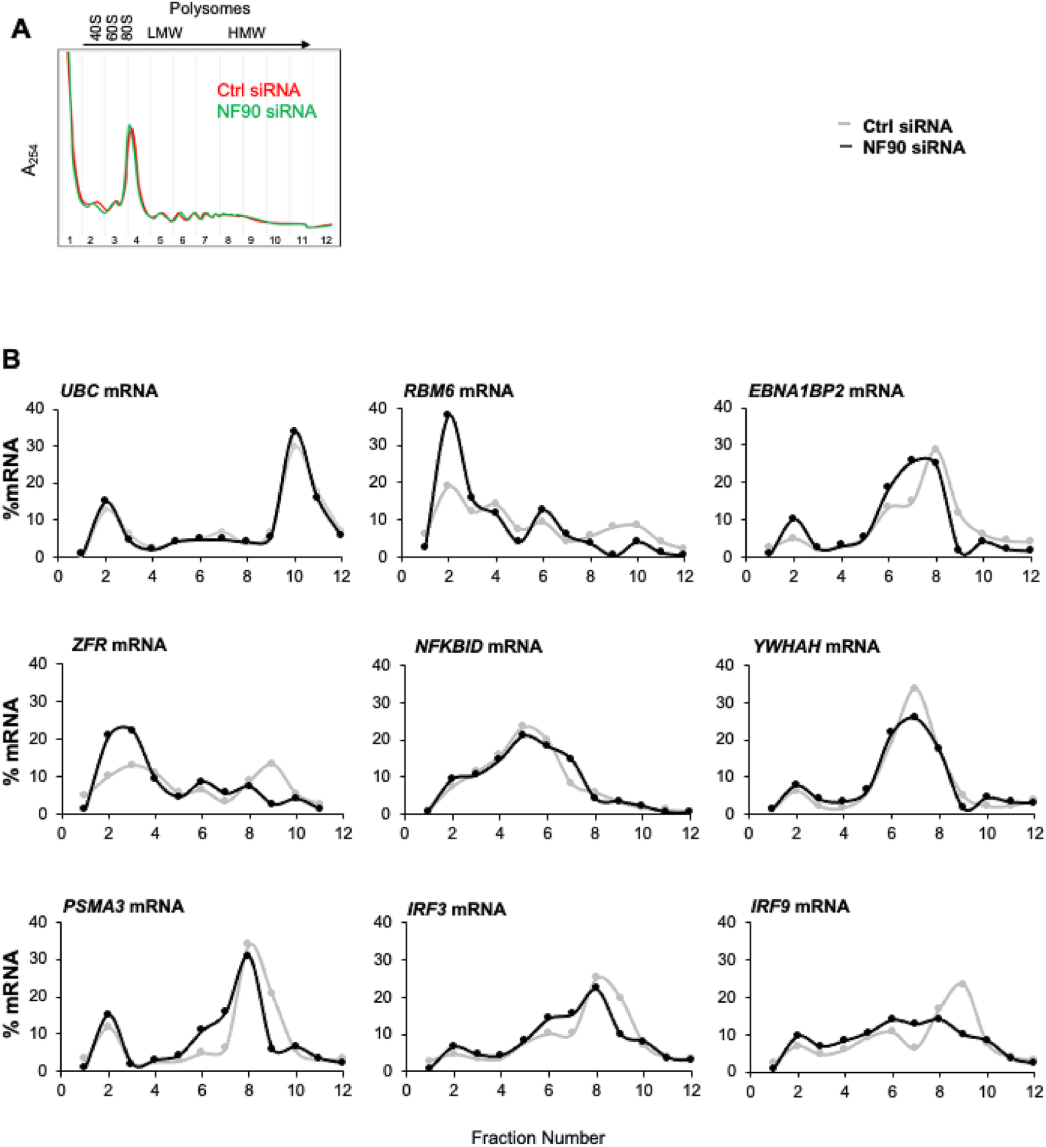
NF90 regulates mRNA translation. Forty-eight hours after transfection of HEK-293 cells with either Ctrl siRNA or NF90 siRNA, lysates were prepared and fractionated through sucrose gradients into 12 fractions. **(A)** 40S and 60S, small and large ribosome subunits, respectively; 80S, monosome; LMW and HMW, low- and high-molecular weight polysomes, respectively (see ‘Materials and methods’ section for details). **(B)** The relative distribution of each NF90 target mRNA and housekeeping *UBC* mRNA was studied by RT-qPCR analysis of RNAs in each of fraction, and plotted them as a percent of the total RNA in the entire gradient. Data are representative of three independent experiments.

We then turned our attention to the possibility that NF90 might influence the translation of some target mRNAs. We specifically assessed whether the association of the mRNA with polysomes might be influenced by NF90 levels (Fig. 6). Cells expressing control or silenced NF90 levels (prepared as described in Fig. 4) were collected and the cytoplasmic components were fractionated by centrifugation through linear sucrose gradients (10–50%), whereupon fractions were collected, and RNA was isolated from each fraction. Fractions 1 and 2 contained free RNA, not associated with ribosomal components; fractions 3–5 contained ribosomal subunits (40S, 60S) and monosomes (80S); and the remainder contained low-molecular-weight (LMW, fractions 6–8) and high molecular-weight (HMW, fractions 9–12) polysomes. As shown, silencing NF90 did not change the global distribution profiles (Fig. 6A). In this analysis, a shift in the direction of larger polysomes is interpreted to reflect more active translation for a given mRNA, while a shift to smaller polysomes is associated with reduced translation.

We then performed RT-qPCR analysis of the same subset of mRNAs in Fig. 5. We included analysis of the distribution of *UBC* mRNA, encoding the housekeeping protein Ubiquitin C, as its association with polysomes does not change in an NF90-dependent manner. The relative distribution of a specific target mRNA in each fraction was plotted as the relative distribution (%) of that mRNA in each fraction calculated over the total levels of that mRNA in the entire gradient. For the majority of the mRNAs tested (Fig. 6B), cells with lower NF90 levels showed reduced presence in polysomal fractions. For example, for *IRF3* and *IRF9* mRNAs, the peaks of abundance (fractions 8 and 9) were reduced after NF90 silencing. Similar shifts towards lighter fractions (less translation) were observed for other mRNAs after silencing NF90, including *RBM6*, *PSMA3* and *ZFR* mRNAs. These data suggest that NF90 promotes the translation of several NF90 target mRNAs in unstimulated HEK-293 cells.

### NF90 is downregulated in PBMC of patients affected by SARS-CoV-2 infection

Considering that some of the identified NF90 targets encode proteins with direct roles in fighting the SARS-CoV-2 infection, we further explored the binding of NF90 to *IRF3* and *IRF9* mRNAs in immune cells and then studied the impact of NF90 on their expression levels in peripheral blood mononuclear cells (PBMCs) purified from a small heterogenous group of patients infected with the SARS-CoV-2 virus.

**Figure 7:**
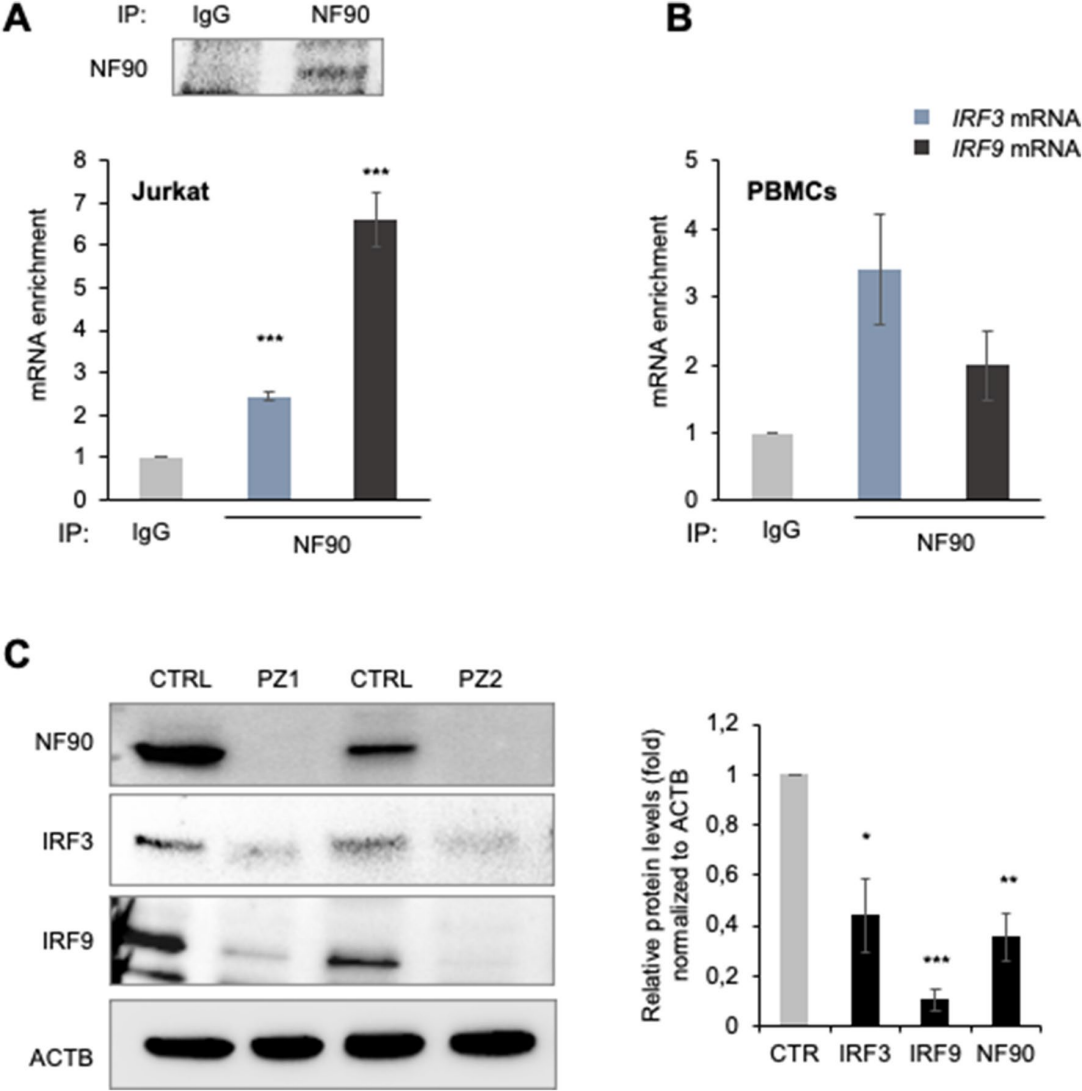
NF90 and IRF3/9 levels decline during COVID-19 infection. **(A,B)** NF90 RIP analysis in Jurkat cells and PBMCs; NF90 or IgG antibodies were used for RIP from Jurkat lysates **(A)** or PBMC lysates **(B)**, and the presence of NF90 in the IP was assessed by Western blot analysis **(A)**. The levels of target *IRF3* and *IRF9* mRNAs in the RIP samples were measured by RT-qPCR analysis and the levels of enrichment of these mRNAs in NF90 IP relative to IgG IP were calculated after normalization to *ACTB* mRNA levels in each IP. **(C)** In PBMCs purified from healthy control (CTRL) and from patients (PZ) infected with SARS-CoV-2, the levels of NF90, IRF3, and IRF9 were assessed by Western blot analysis (left panel) and subsequently quantified using ImageJ (right panel). The levels of ACTB were included to monitor loading in Western blots analyses. Data are the means and standard deviation (+ SD) from at least three independent experiments. *, P < 0.05; ***, P < 0.005.

We first assessed the binding of NF90 to *IRF3* and *IRF9* mRNAs by RIP analysis using Jurkat cells (an immortalized T lymphocyte line), which are known to express high levels of both IRF3 and IRF9^32^. NF90 RIP analysis in Jurkat cells was performed as described in Fig. 2; the levels of NF90 protein present in the IP samples were examined by western blot analysis (Fig. 7A, upper), and purified RNAs were analyzed by RT–qPCR analysis employing specific primer pairs as described above and in^28^. As shown, *IRF3* and *IRF9* mRNAs were highly enriched in NF90 IP as compared to IgG IP samples (Fig. 7A). To extend this analysis to human primary cells, we then performed RIP analysis using purified PBMCs from healthy human donors (Materials and Methods). Similar to Jurkat and HEK-293 cells, NF90 also associated with target *IRF3* and *IRF9* target mRNAs in human PBMCs (Fig. 7B).

Finally, we analyzed the levels of NF90, IRF3, and IRF9 in a small subset of patients infected with the SARS-CoV-2 virus as compared with healthy controls. Western blot analysis followed by quantification by densitometry using ImageJ revealed drastically reduced levels of NF90 in PBMCs purified from SARS-CoV-2-infected patients (PZ) (Fig. 7C), and also strongly reduced levels of the two type-I interferon response proteins IRF3 and IRF9 levels in the same patients (Fig. 7C). These data indicate that future studies are warranted to analyze the relevance of NF90 in the immune response against SARS-CoV-2 infection and, hopefully to identify new therapeutic strategies in combatting this virus.

## Discussion

In this study, we report a comprehensive analysis of RNA targets of NF90 in HEK-293 cells using iCLIP. We found that many NF90 target mRNAs encode proteins with roles in eukaryotic and viral RNA metabolism (Fig. 1), including IRF3 and IRF9, which are directly implicated in the response to SARS-CoV-2 infection. In unstimulated HEK-293 cells, NF90 influenced only slightly the stability of some target mRNAs, but appeared to have a clearer effect on translation, as silencing NF90 decreased the size of polysomes associated with many NF90 target mRNAs and lowered the levels of the encoded proteins. These findings expand our previous knowledge of the functions of NF90 on target RNAs^13,17–19^.

One of the few NF90 targets that appeared to be degraded in an NF90-dependent manner was *YWHAH* mRNA, which showed increased stability after silencing NF90 (Fig. 5). YWHAH belongs to the 14–3-3 protein family and has been implicated in a variety of neurological disorders including Parkinson and Creutzfeldt-Jakob disease^33^. A deeper analysis to understand the involvement of NF90 and YWHAH in neurodegeneration is warranted. The small but significant change in the stability of *IRF9* mRNA also deserves further attention, particularly to elucidate the molecular mechanisms mediating this influence. Even more importantly, future studies should determine whether the influence of NF90 on the stability of target mRNAs changes in response to stimuli that affect the immune and nervous systems.

For other target mRNAs examined, particularly *IRF3, IRF9, RBM6,* and *ZFR* mRNAs (Fig. 6), NF90 modified their association with polysomes, an indirect measure of their translation efficiency. Here too, the precise mechanistic effect needs to be analyzed in future studies. As with other RBPs, changes in the translation of NF90 target mRNAs could be mediated through either cooperation or competition with RBPs and/or microRNAs that can influence their translation. An example of cooperation of NF90 with miR-15a leading to the repression of *BAFF* mRNA translation was recently reported^30^. Identifying the specific factors that modulate translation of NF90 target mRNAs found in this study will need to be undertaken individually for each mRNA and its cellular context, as the collection of mRNA-binding factors (proteins and RNAs) associating with a given mRNA varies depending on the mRNA sequence, the cell type, and the growth conditions.

IRF3 and IRF9 deserve particular attention, as these transcription factors are directly involved in the regulation of the type I interferon (IFN) response against viral infection^34,35^. Interestingly, it was recently reported that SARS-CoV-2 infected patients produce aberrant levels of type I IFNs and alterations in the function of IRF proteins^25^. Indeed, several SARS-CoV-2 proteins, including proteins of the viral non-structural proteins (nsp) and the nucleocapsid (N) protein, have been reported to inhibit type I IFN activation and the corresponding responses through regulation of IRF proteins^36^. For example, Wang and colleagues found that the SARS-CoV-2 nsp12 protein attenuates type I IFN induction by inhibiting IRF3 nuclear translocation^37^. Given the potential relevance to the COVID-19 pandemic, we sought to confirm the association of NF90 with *IRF3* and *IRF9* mRNAs in PBMCs and the steady-state levels of IRF3, IRF9 and NF90 in PBMCs purified from SARS-CoV-2-positive patients and healthy controls. Although in PBMCs and immortalized lymphocytes NF90 associated with *IRF3* and *IRF9* mRNAs, there was a striking loss of NF90 in PBMCs from SARS-CoV-2-positive individuals associated with a decline in IRF3 and IRF9 levels (Fig. 7). Interestingly, this regulation appears to vary across tissues; for example, RNA samples extracted from nasal swabs revealed that persons with SARS-CoV-2 had significantly elevated IRF3 and IFN relative to uninfected patients^38,39^. The role of NF90 in establishing a type I IFN program upon dsRNA stimulation was described by Watson and colleagues in cervical cancer cells^40^; the authors found that NF90 modulated gene expression programs after dsRNA stimulation, and observed that IRF1 was less induced when NF90 levels were low^40^, in line with what we observed for IRF3 and IRF9. Taken together, our findings underscore the relevance of future studies to systematically analyze the impact of NF90 on IFN and IRF proteins, which are deeply implicated in the devastating COVID-19 pandemic.

The role of NF90 in the regulation of genes involved in the immune response was previously demonstrated in other different contexts. For example, NF90 was shown to bind a subset of RNAs containing AU-rich elements in the 3'UTR, including *IL2* mRNA during T cell activation^9^. We also reported the role of NF90 in modulating several cytokines in response to *Plasmodium falciparum* antigens^17^ and the role of NF90 as a repressor of the translation of the cytokine BAFF in THP-1 cells^18^. Additionally, we confirmed that NF90 binds A- and U-rich sequences present in target mRNAs as previously reported^27^ (Fig. S1). Other RBPs, including AUF1 and HuR also preferentially bind AU-rich target RNAs^41^; thus, it is likely that other RBPs compete or cooperate with NF90 to define the post-transcriptional outcome of specific transcripts.

Besides binding and regulating mammalian mRNAs, NF90 and its isoforms also bind viral genomes (RNA and DNA). Depending on the virus, NF90 can act as either a negative or a positive regulator of the expression of viral genes^42^. For example, NF90 had antiviral activity by antagonizing the inhibitory role of the nonstructural protein 1 (NS1) on PKR phosphorylation during influenza A infection^43^.

In closing, using comprehensive, high-throughput approaches, we have identified numerous NF90 target mRNAs, and found that the encoded proteins are predicted to influence mammalian and viral RNA metabolism. We confirmed earlier observations that NF90 can influence mRNA turnover and translation, and characterized in molecular detail how NF90 affects the stability and translation of *IRF3* and *IRF9* mRNAs, two key factors in the IFN response. Our findings set the stage for a deeper investigation of the impact of NF90 on the immune response during SARS-CoV-2 infection, and a search for therapeutic modulators of NF90 function.

## Materials and methods

### Cell culture and modulation of NF90 levels

Human Embryonic Kidney 293 (HEK-293) cells were cultured in Dulbecco's modified Eagle's medium (DMEM, Invitrogen Life Technologies) supplemented with 10% fetal bovine serum and antibiotics. HEK-293 cells expressing low levels of NF90 (Ctrl or NF90 siRNA) and high levels of NF90 were generated (pcDNA3-MYC-NF90 expression vector) using the Lipofectamine 2000 transfection reagent (Invitrogen Life Technologies) following the manufacturer’s instructions. Jurkat cells (human T lymphocyte cell line) were cultured in RPMI 1640 medium supplemented with 10% fetal bovine serum and antibiotics.

### RNA isolation, reverse transcription (RT)-quantitative (q)PCR analysis

Total RNA was isolated using the TRIzol isolation reagent (Thermo Fisher) following the manufacturer’s protocol and RNA integrity was verified by agarose gel electrophoresis. First-strand cDNA synthesis was performed using Maxima reverse transcriptase (Thermo Fisher) and random hexamers. The generated cDNA was diluted tenfold and used as a template for quantitative (q)PCR analysis using SYBR Green mix (Kapa Biosystems). Relative levels of RNA were calculated using the 2^−ΔΔCt^ method and the levels of β-actin (*ACTB*) mRNA were used for normalization. Gene-specific primer pairs are listed in Table 1. RNA was isolated from PBMCs using the M-PER Mammalian Protein Extraction Reagent (Invitrogen Life Technologies).

**Table 1:** List of primers used.

Gene name	Primer Sequences (5’–3’)
**RT-(q)PCR primers**	
PIN1	FW: CTGGTGAAGCACAGCCAGT
	RV:CTCCCGACTTGATCTTCTGG
EPS15L1	FW:GGCTTTGCAGACTTCACCTC
	RV: GCCAGTTCCAGGTCCTCC
IRF3	FW: GTGGCCTGGGTGAACAAGAG
	RV: ATTCCGAAATCCTCCTGCTGT
FGF23	FW: CTACCACCTGCAGATCCACA
	RV: ATCACCACAAAGCCAGCATC
IL27RA	FW: TGTGGGTATCAGGGAACCTC
	RV: TCCAACCCAGAACCAGACTT
RBFOX1	FW: ACACGTCTGGAGGAGACAGC
	RV: AGGGGTAATGCTTGGTGATG
RPS3	FW: TGAGGTGCGAGTTACACCAA
	RV: ACAGCAGTCAGTTCCCGAAT
RGS14	FW: GCAGACCTGTGACATCGAAG
	RV: GAAGTACCAGGTCCTCTTTCC
PSMB11	FW: CCCACAGAGCTGCAGAAGAT
	RV: CTGCTTCTCACACCGTCTCA
RBM6	FW: AGCCCTATGTCCGCCTTACT
	RV: GGCGGATCAAGGTTCTGTAA
PAF1	FW: TGGAACAACTGTGGAACCTG
	RV: TCACTGCCCTCCTTCTCACT
TWIST1	FW: GGCTCAGCTACGCCTTCTC
	RV: TCCATTTTCTCCTTCTCTGGA
FGFR3	FW: ATCCTCGGGAGATGACGAA
	RV: CAGCAGCTTCTTGTCCATCC
MAST1	FW: AATTTCTCGATGCCCTCCTT
	RV: AGGATGAGGCTTTTCCGATT
MED24	FW: GAGAAAACCCTCAGCAGCAC
	RV: CTCGATAAGGGCAGTCCAAG
DNAAF5	FW: TCTGCTGCTCAGTAGCCTCA
	RV: TTCAGGTCCTGCTCCTCATT
YWHAH	FW: TATGAAGGCGGTGACAGAGC
	RV: ATGCTGCTAATGACCCTCCA
TMEM107	FW: CCGGTTTCCTCTCAGGAGTC
	RV: TACGTAGTGCACTCCCAACG
IL13RA1	FW: TCCCAGTGTAGCACCAATGA
	RV: AGGGAGCCAAGAACACTTCA
CLEC16A	FW: CTCCACAGCCCAGAGTCC
	RV: TGGGCTTAGAGTCTGCTTCC
CDC27	FW: TCGCCCTGATGAATTTCTCT
	RV: GTGTGTCATCCGCATCTGTC
IRF9	FW: GAAAACTCCGGAACTGGGTG
	RV: ATCCGGAACATGGTCTTAGCT
GABARAPL3	FW: CAAAGAGAAGGGAGCACAGG
	RV: AGAAAGGTCGGTCAGGAGGT
CDK14	FW: GATCAGGCTGCAGGAAGAAG
	RV: AGTGTCAGCGTCTCCTTGGT
PTCH1	FW: GACCGGGACTATCTGCACC
	RV: AGTCTCTGAAACTTCGCTCTCA
EBNA1BP2	FW: TCCGATGAATCCCTTGTCACA
	RV: CCCCTCTAGCACGACATTGA
HIST1H4H	FW: AGTTTTCCTGTGGCTCCTGA
	RV: TAGGCAGCATCTCCATAGCA
MORF4L1	FW: GAAATGGAGATGGTGGCAGT
	RV: GCCACGGTTTTAGCTCTTCA
PSMD11	FW: CATCGACATCCTCCACTCCA
	RV: CCAGTCTTTGCCAGGAGAGA
AGO4	FW: AAAACGGCCTCGTAGAGTCA
	RV: CCGTCCAATTGGTAGTGGAT
SF3B3	FW: CCTGCAGCCATGTTTCTGTA
	RV: CCACGGGAAACAACAATTTC
NFKBID	FW: AGCTCACATGCTGGCTTTG
	RV: GCCCTTATGCTCACGAATGT
PSMA3	FW: GTGGTATAGGGGAAGCGCTC
	RV: CGTCAGGAGAGAATGTAGAGGC
CDKN2B	FW: ACTAGTGGAGAAGGTGCGAC
	RV: TCATCATGACCTGGATCGCG
DDX39B	FW: TGATGTGCAGGATCGCTTTG
	RV: CTGGTGTCCTCTCCTGAAGG
ATP5B	FW: CCCAGCTCAGCTCTTACTGC
	RV: TTTTGGCGAAGGAGATGTTT
ADAM10	FW: CCTACGAATGAAGAGGGACACT
	RV: CAGACCCATGGCTAAAACTTCC
LARP4	FW: CAGTTGCCAACATGGAAGAA
	RV: TGGTCTCACTTTCTCACCCTTC
ZFR	FW: ACTGCACACACAGCAACTGAC
	RV: CAGGAGCTGTGGACCTTACAT
NF90	FW: GCTGTGTCCGACTGGATAGAC
	RV: AGCCCCTTCTTTACTGTCGTC
BACT	FW: CATGTACGTTGCTATCCAGGC
	RV: CTCCTTAATGTCACGCACGAT
LINC00324	FW: AGGACACGTGGTCTGCTACC
	RV: TTCCGTAACCTGGGATCTTG
XIST	FW: ATGCCTGGCACTCTAGCACT
	RV: CAAAGGCACACACGAAAGAA
AJ003147.9	FW: GTCGCTACAGGGGCATAAAT
	RV: CTGAAATGTCCTCCTTTGC
MALAT-1	FW: TGGGGGAGTTTCGTACTGAG
	RV: TCTCCAGGACTTGGCAGTCT
**Biotin pull down (T7:AGTAATACGACTCACTATAGGG)**	
Biot-ZFR-1	FW: CTGTTCTCCGCTGAGGAGGA
	RV: GGGCTCGGGCTGCTGCTGCTGA
Biot-ZFR-2	FW: AGCCCATGATTCCCATATGCC
	RV: GTCGAGTTTGCTGGGCTTGA
Biot-ZFR-3	FW: CTCGACAAGTGACAGCCATA
	RV: AACAACATTTGGTTCTGTTG
Biot-ZFR-4	FW: GTTGTTAGCCAAGCTACTTCT
	RV: TTCTTCTCGTCTTCGCCAGTA
Biot-ZFR-5	FW: GAAGAAGAGGAGCGTTGGAGA
	RV: TCGAATAATTGGAGATGTCA
Biot-ZFR-6	FW: ATTCGAGAAGAGAACATGAGGG
	RV: GAAGATTTACAGACACTTT
Biot-ZFR-7	FW: AAAGTGTCTGTAAATCTTC
	RV: CTCTGCAGTCTCACGTTACAA
Biot-ZFR-8	FW: TTGTAACGTGAGACTGCAGAG
	RV: ACTGTTGTTAGTTGTACAATG
Biot-ZFR-9	FW: AACTAGCCCTTAATTATGG
	RV: TAAATTTGAATATGATTTTACTG
Biot-YWHAH-1	FW: ACAGCGCGCGGGGCGAGCCA
	RV: CATGTCGCTCGCGGCTCGGCCT
Biot-YWHAH-2	FW: AGCGACATGGGGGACCGGGAGCA
	RV: TCAGTTGCCTTCTCCTGCTT
Biot-YWHAH-3	FW: AACTGAAGATCCTTCAGGTC
	RV: CATTTTTGCATGCACAAGATG
Biot-YWHAH-4	FW: GCAAAAATGAATTCACCCCT
	RV: TAATATCCCCAAAGCAGCAT
Biot-PSMA3-1	FW: ACTAGTTTGCGGCATCCTG
	RV: GCTCATCGTGCTAAACCCAA
Biot-PSMA3-2	FW: AGCACGATGAGCTCAATCGG
	RV: TAAATGTTACATATTATCATC
Biot-PSMA3-3	FW: ATGTAACATTTACTCCAGCAT
	RV: TATAAGACAAAAATTTAATG
Biot-IRF3-1	FW: AAAGAATGATAAAGTTGGTTTT
	RV: GGTCCGGCCTACGATGGAAGGT
Biot-IRF3-2	FW: CGGACCATGGGAACCCCAAAGC
	RV: CACAAACTCGTAGATTTTATGTG
Biot-IRF3-3	FW: AGTTTGTGAACTCAGGAGTTGG
	RV: TGCTGGAAGACTTGGCGGCCC
Biot-IRF3-4	FW: TTCCAGCAGACCATCTCCTGCC
	RV: CGTGAAGGTAATCAGATCTGGG
Biot-IRF3-5	FW: CCAGATCTGATTACCTTCACGGA
	RV: TCATAGCAGGAACCAGTTTATT
Biot-IRF9-1	FW: GGAGAGATCAGCCGCCCAGC
	RV: CTGAGTTGCTGTCCAGGCT
Biot-IRF9-2	FW: GCAACTCAGGATGGCATCAG
	RV: CCTGGCTGGCCAGAGACGATT
Biot-IRF9-3	FW: GAATCGTCTCTGGCCAGCCA
	RV: CTTGGGGAACAGCACCTGCTCC
Biot-IRF9-4	FW: GGAGCAGGTGCTGTTCCCCAAG
	RV: ATGGGTCCCCCAGGCTCTACACCAG
Biot-IRF9-5	FW: AGCCATTAGCCTGGGGGACCCAT
	RV: TTCCTCTTTCAATAAAA
Biot-neg (BAFF 3'UTR)	FW: AAGTGCCCACATCTCTAGGACA
	RV: GTATATGGATACTATTTTCAGCAAAATTGTTTC

### RNA sequencing analysis

RNA sequencing (RNA-seq) and RNA-seq analysis were performed by Quick Biology. Briefly, RNA integrity was first assessed by using the Agilent Bioanalyzer 2100. Only samples with clean rRNA profiles were used for subsequent steps. Libraries for RNA-seq analysis were prepared according to KAPA Stranded RNA-Seq Kit with RiboErase (KAPA Biosystems) system. Paired-end sequencing was performed using Illumina HighSeq 4000 instrument (Illumina Inc.).

### RIP (ribonucleoprotein immunoprecipitation) analysis

RIP analysis was performed as previously described^28^. Briefly, PEB buffer (10 mM Hepes, 100 mM KCl, 5 mM MgCl_2_, 25 mM EDTA, 0.5% IGEPAL, 2 mM DTT, 50 U/ml RNase out and protease inhibitors) was used to prepare lysates of HEK-293 cells and M-PER Mammalian Protein Extraction Reagent (Thermo Scientific) was used to prepare lysates of PBMCs. After preclearing (10 min on ice followed by 15 min centrifugation), 500 μg of lysates were incubated (4 h, 4 °C) with a suspension of protein-A Sepharose beads (GE Healthcare) precoated overnight with 5 μg of a NF90 specific antibody (BD Transduction Laboratories) or normal mouse IgG (Santa Cruz Biotechnology) used as a control. Subsequently, the beads were washed with NT2 buffer (50 mM Tris–HCl pH 7.5, 150 mM NaCl, 1 mM MgCl_2_, 0.05% IGEPAL) and then treated with RNase-free DNase I (15 min, 30 °C). RNAs that associated with this NF90 were then isolated using TRIzol and their levels of enrichment were assessed by RT-qPCR analysis using the primers listed in Table 1. Target mRNA levels were first normalized to *ACTB* mRNA levels, and then enrichments were calculated as the relative abundance of a target mRNA in NF90 IP as compared to IgG control IP.

### Western blot analysis

HEK-293 and PBMC cell lysates were prepared with RIPA buffer (10 mM Tris–HCl [pH 7.4], 150 mM NaCl, 1% NP-40, 1 mM EDTA, 0.1% SDS, 1 mM dithiothreitol); 20 μg protein aliquots from each sample were denatured and size-fractionated by electrophoresis through SDS-containing, 4–12% gradient polyacrylamide gels (Thermo Fisher Scientific), SDS-PAGE. The proteins were then transferred onto nitrocellulose membranes using Trans-Blot Turbo Transfer System (Biorad). Full-length membranes or membranes cropped based on molecular weight were incubated for 16 h with the appropriate primary antibodies recognizing target proteins NF90 (BD Transduction Laboratories) EPS15L1 (OriGene Technologies), RGS14, EBNA1BP2 (Novus Biologicals), PSMD11, DDX39B, RBM6, NFKBID, ZFR, YWHAH, PSMA3, IRF3, IRF9 (Abcam), ACTB (Santa Cruz Biotechnology). After incubation with appropriate secondary antibodies conjugated with horseradish peroxidase (HRP) (Kindle Biosciences), protein signals were detected by enhanced chemiluminescence (Euroclone). Images of uncropped membrane are present in Supplemental Fig. S5.

### Analysis of polysome gradients

To identify mRNAs engaged in translation, cells were incubated with 0.1 mg/ml cycloheximide for 10 min and lysates were prepared from HEK-293 cells (Ctrl or NF90 siRNA). Cytoplasmic extracts were then loaded on top of a linear sucrose gradient [10–50% (w/v)] and size-separated by ultracentrifugation as previously reported^44^. Fractions were collected using a density gradient fractionation system and monitored by optical density measurement (A_254_) (Brandel). The RNA in each fraction was isolated using TRIzol reagent and reverse-transcribed; the relative levels of a given mRNA in a specific gradient fraction were calculated using qPCR analysis with the primers listed (Table 1) and represented as a percentage of the total mRNA on the gradient.

### Synthesis of biotinylated fragments and pull-down assay

To synthesize biotinylated RNA, gene-specific forward PCR primers containing the T7 RNA polymerase promoter sequence were used. After purification of the PCR products, the following biotin-RNAs (bi-RNA) were synthetized using MaxiScript rT7 kit (Ambion): bi-ZFR, bi-YHAW, bi-PSMA3, bi-IRF3, and bi-IRF9. A fragment of *BAFF* 3'UTR RNA that was previously demonstrated not to bind with NF90 was used as negative control^30^. Bi-RNA pulldown assay was performed as previously described^45^ using 200 µg of whole-cell lysates from HEK-293 cells. Briefly, cells were lysed using PEB buffer (10 mM Hepes, 100 mM KCl, 5 mM MgCl_2_, 25 mM EDTA, 0.5% IGEPAL, 2 mM DTT, 50 U/ml RNase out and protease inhibitors) and incubated 30 min with 1 μg of purified biotinylated transcripts for 1 h at 25ºC. Complexes were isolated with Streptavidin-coupled Dynabeads (Invitrogen) and the proteins present in the pulldown material were detected by Western Blot analysis.

### Analysis of mRNA stability by actinomycin D assays

Actinomycin D (Act D; 2.5 µg/ml) was used to inhibit de novo transcription in cultured HEK-293 cells. Cells were then harvested between 1 and 6 h and total RNA extracted using TRIzol reagent (Invitrogen Life Technologies) according to the manufacturer's instructions. RT-qPCR analysis was then performed using *ACTB* mRNA levels as normalization control. mRNA decay from Act D assays were processed using Prism8.2.1 software.

### iCLIP procedure

iCLIP was performed as previously descried^46^. Briefly, HEK-293 cells were cultured in 10-cm plates and stably transfected with pcDNA3-MYC-NF90. After irradiation with 150 mJ/cm^2^ at 254 nm in a UV Stratalinker 2400 (Stratagene) on ice, cells were collected and snap-frozen at -80 °C. Cells were lysed using lysis buffer [50 mM Tris–HCl, 100 mM NaCl, 1% Igepal CA-630, 0.1% SDS, 0.5% sodium deoxycholate] supplemented with Protease and RNase inhibitors, sonicated and treated with 2 μl of RNase I (Ambion) diluted 1:150. Lysate samples were then immunoprecipitated using anti-MYC antibody overnight at 4 °C with rotation. For library preparation and visualization, the RNA was dephosphorylated, an adapter was ligated at the 3’ end and it was radioactively labeled at the 5’ end with γ-^32^P-ATP. The protein-RNA complexes were separated by SDS–PAGE and then isolated from a nitrocellulose membrane according to the expected size; protein digestion was performed using proteinase K and RNA was isolated. RNA fragments were reverse-transcribed using primers that introduce two cleavable adapter regions and barcode sequences. Circularization of the cDNA was carried out followed by linearization using the restriction enzyme BamHI. Linearization generated appropriate templates for PCR amplification optimized to avoid the generation of secondary products. Libraries were finally sequenced using high-throughput RNA-seq analysis.

### iCLIP pipeline

Raw reads were obtained using bcl2fastq from the NextSeq500 run. Adapter and low-quality bases (< Q30) were trimmed using *cutadapt*^47^ and *sickle*^48^. More than 75 million reads were retained. The overlapping forward and reverse reads were merged using *Pandaseq* to generate longer and complete sequences^49^. The randomized 5-bp molecular barcodes were extracted using *UMI-tools*^50^ and the reads were demultiplexed using *reaper*^51^. A total of 2,518,715 reads were obtained and demultiplexed reads were then mapped to hg19 using *STAR* aligner^52^. In more details, the demultiplexed reads were processed following the eCLIP ENCODE pipeline described in https://www.encodeproject.org/pipelines/ENCPL357ADL/: (i) reads were mapped to human specific version of *RepBase* (accessed 18 set 2018) with the STAR alignment tool to remove repetitive elements; (ii) unmapped reads from the first STAR run were than sorted and aligned to the human genome (hg19 with Gencode V19 annotations); (iii) mapped reads were then deduplicated (to remove PCR duplicates) with the command *umi_tools dedup*. Finally, (iv) output BAM files were then merged into an unique indexed and sorted BAM file. Identification of Crosslinked sites was performed with iCount, a Python module (Python software 4.10.2), and associated command-line interface (CLI), which provides all the commands needed to process protein-RNA iCLIP interaction data and to identify and quantify sites of protein-RNA interactions on RNA^53^. Binding signals were also confirmed with the Clipper tool^54^. Mean coverage of iCLIP data was calculated from different genomic regions using only signal with significant p-value (< 0.05). Motif analysis was performed using HOMER (Hypergeometric Optimization of Motif EnRichment) a suite of tools for Motif Discovery (HOMER software v4.11.)^55^.

Pathway enrichment analysis was conducted with the R library (R software 4.1.2) cluster Profiler [https://bioconductor.org/packages/release/bioc/html/clusterProfiler.html]. Given a vector of genes, the function *enrichGO* returns the enrichment GO categories after FDR control.

### PBMC purification

Peripheral blood samples were collected from adult donors positive for COVID-19 infection and adult healthy donors. All of the human samples used in this study were previously anonymized as required by the Italian Data Protection Code (Legislative Decree 196/2003) and the general authorizations issued by the Data Protection Authority. Ethics Committee approval was deemed unnecessary because, under Italian law, it is only required in the case of prospective clinical trials (Art. 6 and Art. 3 of Legislative Decree 211/2003). However, all of the patients gave their written informed consent to the medical procedures/interventions carried out for routine treatment purposes. Peripheral blood mononuclear cells (PBMCs) were purified from a small heterogenous group of patients infected with the SARS-CoV-2 virus and healthy human donors using Histopaque 1077. About 15 ml of blood was collected from each donor using BD Vacutainer CPT tubes containing 0.1 M sodium citrate and PBMCs were isolated following the manufacturer’s instructions. The isolated PBMCs were then used for Western Blot and RIP analysis.

### Statistical analysis

Statistical significance was determined using two-tailed *t*-tests, as indicated in the Figure legends. Values were considered significant when p < 0.05.

## Supplementary Information

Supplementary Figures.

Supplementary Table S1.

Supplementary Table S2.
