# Supplementary material for: Systematic identification of NF90 target RNAs by iCLIP analysis
Source: Sci Rep. 2022 Jan 10;12:364. doi: 10.1038/s41598-021-04101-1 (PMC8748789; doi:10.1038/s41598-021-04101-1)
Supplement: Supplementary file 1 — Supplementary Figures. [file 41598_2021_4101_MOESM1_ESM.pdf]

Systematic identification of NF90 target RNAs by iCLIP analysis

Valeria Lodde[1,2], Matteo Floris[1], Rachel Munk[2], Jennifer L. Martindale[2], Davide Piredda[5], Catello Mario Panu Napodano[6], Francesco Cucca[1], Sergio Uzzau[1,7], Kotb Abdelmohsen[2], Myriam Gorospe[2], Ji Heon Noh[2,3], and M. Laura Idda[2,4]*

[1]Department of Biomedical Sciences, University of Sassari, Sassari, Italy

[2]Laboratory of Genetics and Genomics, National Institute on Aging Intramural Research Program, National Institutes of Health, Baltimore, MD 21224, USA

[3]Department of Biochemistry, Chungnam National University, Daejeon, Korea

[4]Institute for Genetic and Biomedical Research (IRGB-CNR), Sassari, Italy

[5]Intensive Care Unit, Emergency Department, AOU Sassari, Sassari, Italy

[6]Internal Medicine Unit, ATS Sardegna, Ospedale Civile Alghero, Sassari, Italy

[7]Microbiology and Virology Unit, Diagnostic Department, AOU Sassari, Sassari, Italy

*Corresponding author.

M. Laura Idda; **email:** marialaura.idda@irgb.cnr.it

Supplementary Material

Lodde et al., Supplemental Fig. S1

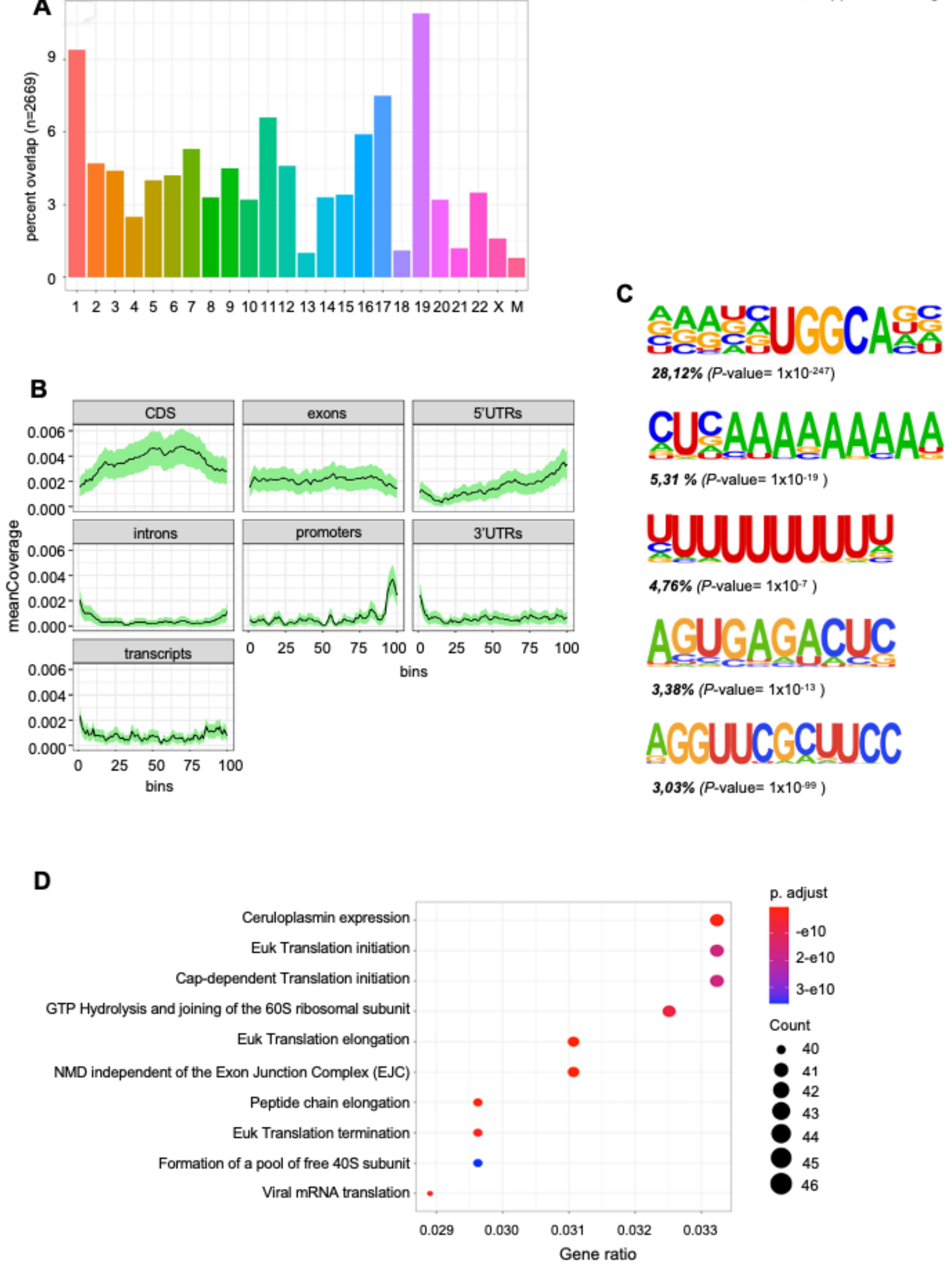

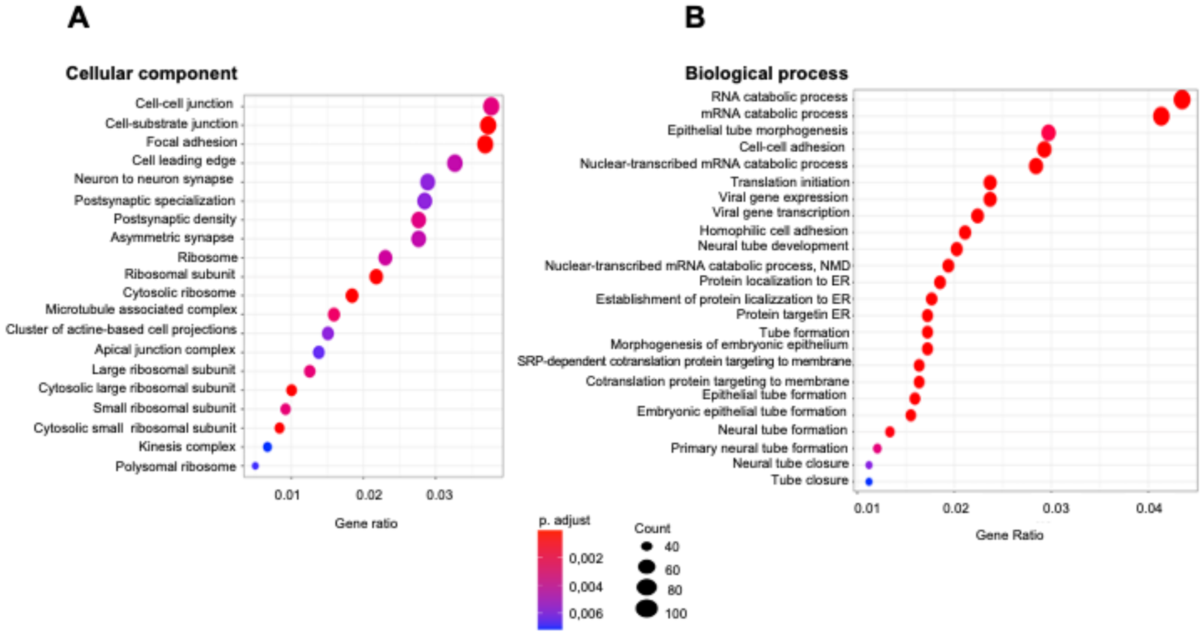
A
Cellular component
Cell-cell junction
Cell-substrate junction
Focal adhesion
Cell leading edge
Neuron to neuron synapse
Postsynaptic specialization
Postsynaptic density
Asymmetric synapse
Ribosome
Ribosomal subunit
Cytosolic ribosome
Microtubule associated complex
Cluster of actine-based cell projections
Apical junction complex
Large ribosomal subunit
Cytosolic large ribosomal subunit
Small ribosomal subunit
Cytosolic small ribosomal subunit
Kinesis complex
Polysomal ribosome
0.01
0.02
0.03
Gene ratio
B
Biological process
RNA catabolic process
mRNA catabolic process
Epithelial tube morphogenesis
Cell-cell adhesion
Nuclear-transcribed mRNA catabolic process
Translation initiation
Viral gene expression
Viral gene transcription
Homophilic cell adhesion
Neural tube development
Nuclear-transcribed mRNA catabolic process, NMD
Protein localization to ER
Establishment of protein licalizzation to ER
Protein targetin ER
Tube formation
Morphogenesis of embryonic epithelium
SRP-dependent cotranslation protein targeting to membrane
Cotranslation protein targeting to membrane
Epithelial tube formation
Embryonic epithelial tube formation
Neural tube formation
Primary neural tube formation
Neural tube closure
Tube closure
0.01
0.02
0.03
0.04
Gene Ratio
p. adjust
0,002
0,004
0,006
Count
40
60
80
100

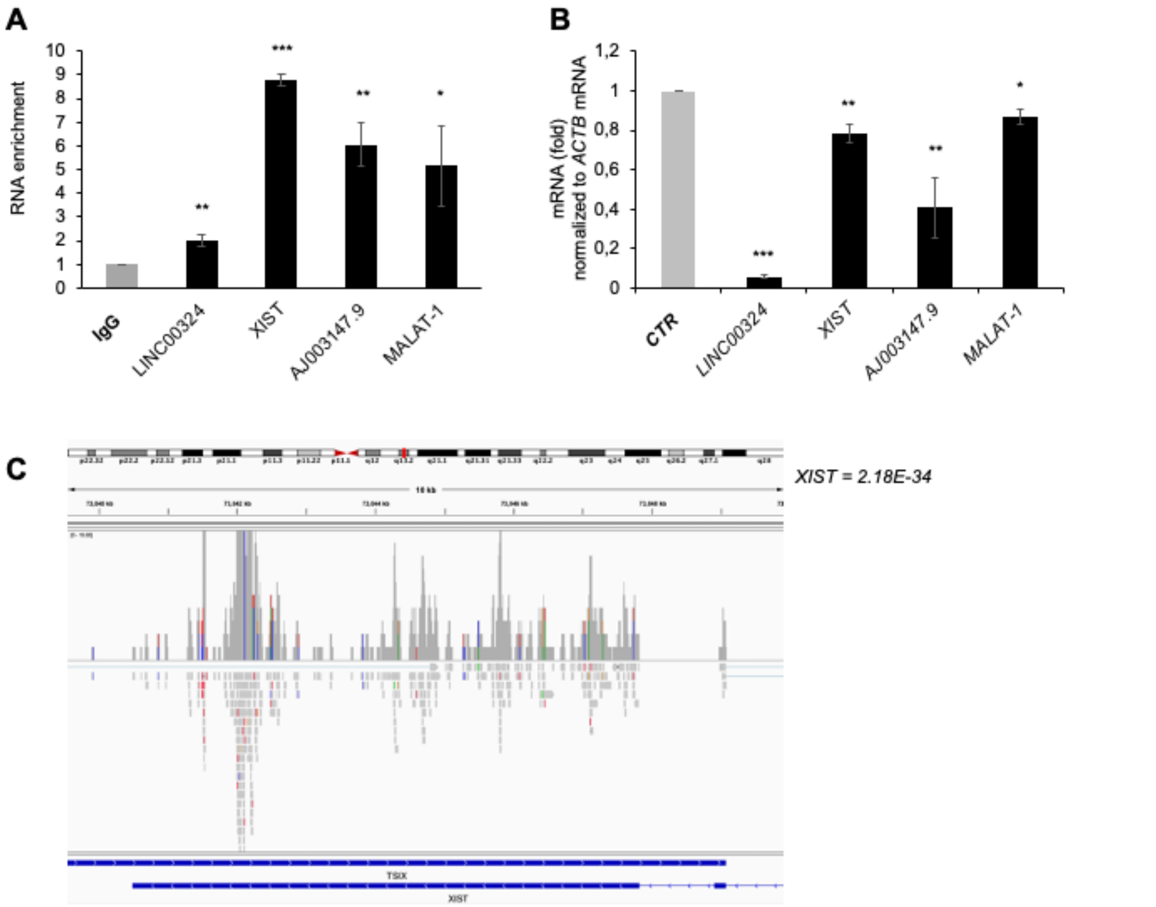
A
RNA enrichment
10
9
8
7
6
5
4
3
2
1
0
***
**
**
*
IgG
LINC00324
XIST
AJ003147.9
MALAT-1
B
mRNA (fold) normalized to ACTB mRNA
1,2
1
0,8
0,6
0,4
0,2
0
***
**
**
*
CTR
LINC00324
XIST
AJ003147.9
MALAT-1
C
XIST = 2.18E-34

A

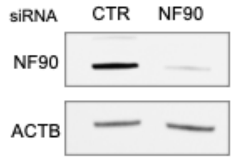

B

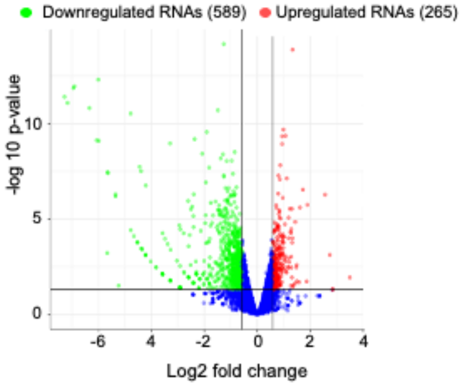

C

RNAs	Log2 FC	*P*-value
SART1	3.3	2.8925E-07
MTRNR2L8	3.0	0.0200592
VSTM2B	2.7	1.4161E-05
SETD7	2.5	1.3352E-14
NES	2.5	9.7234E-05
CEP250	2.5	0.00016994
NEFH	2.5	2.9309E-06
MTRNR2L1	2.5	0.01151693
ILF3	-2.5	7.1562E-17
CNIH2	-2.5	2.8604E-05
HNRNPKP3	-2.6	6.2239E-06
DGCR5	-2.6	6.66E-09
PTH1R	-2.7	5.9518E-06
UNC13D	-2.7	4.6234E-06
DES	-2.7	1.9379E-06
GATA5	-2.8	2.0127E-05
PIGL	-2.8	1.9816E-11
TRPV4	-3.8	2.685E-10
DNASE1L2	-4.2	3.7107E-09

D

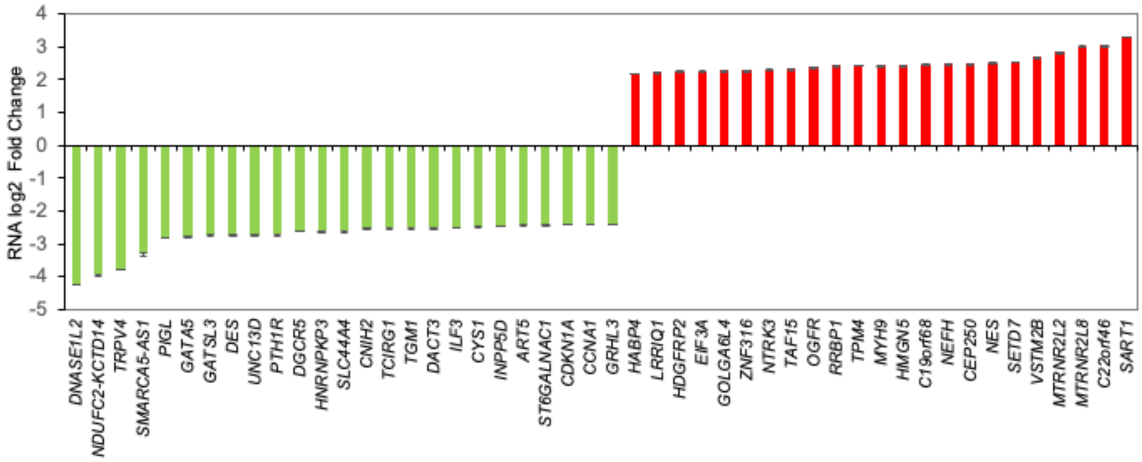

E

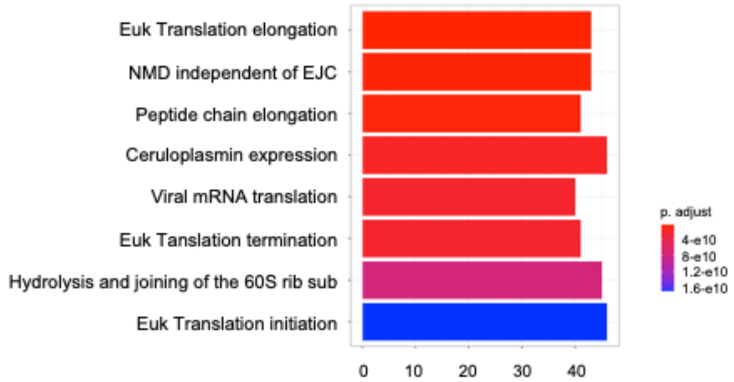

F

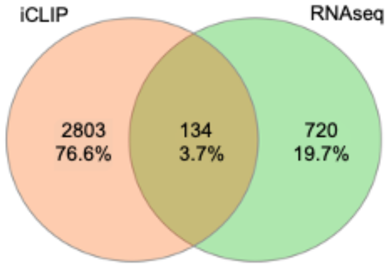

Lodde et al., Fig. 1A

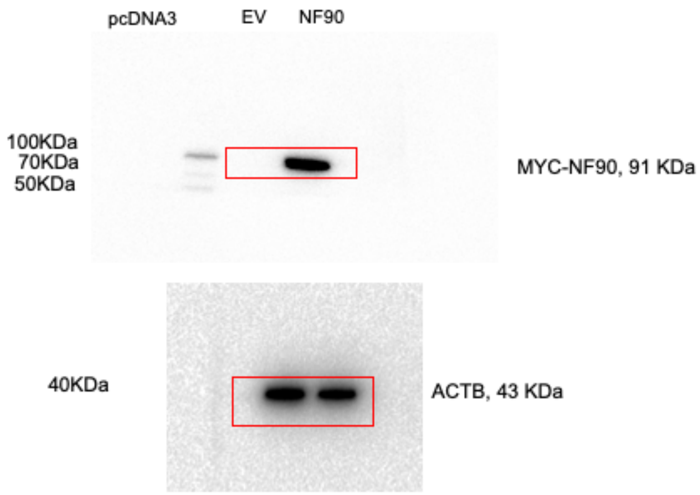

Lodde et al., Fig. 2B

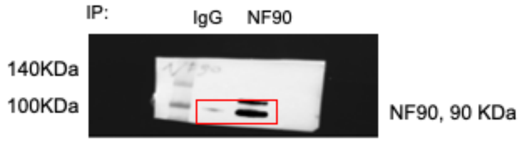

Lodde et al., Fig. 3

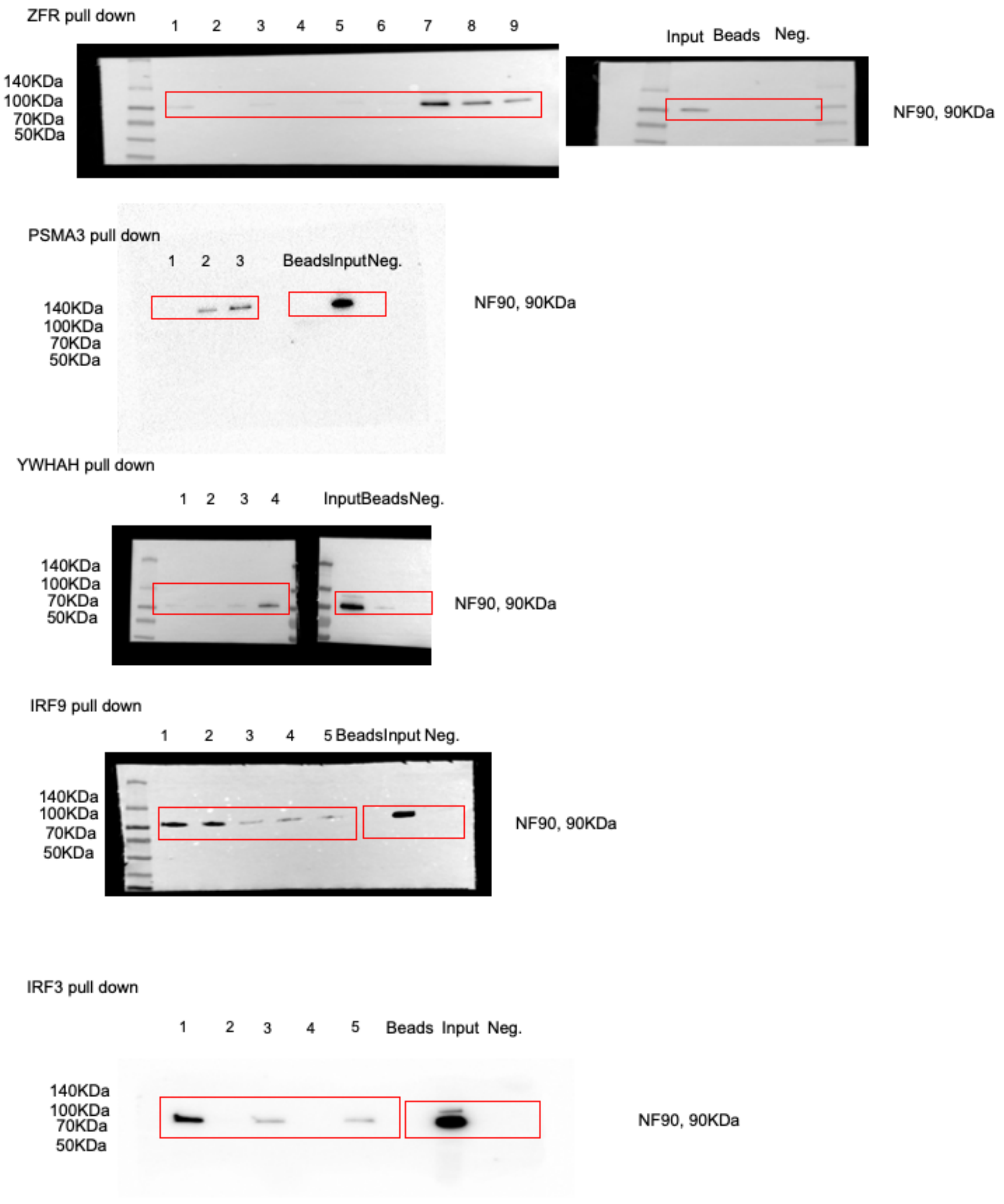

Lodde et al., Fig. 4B

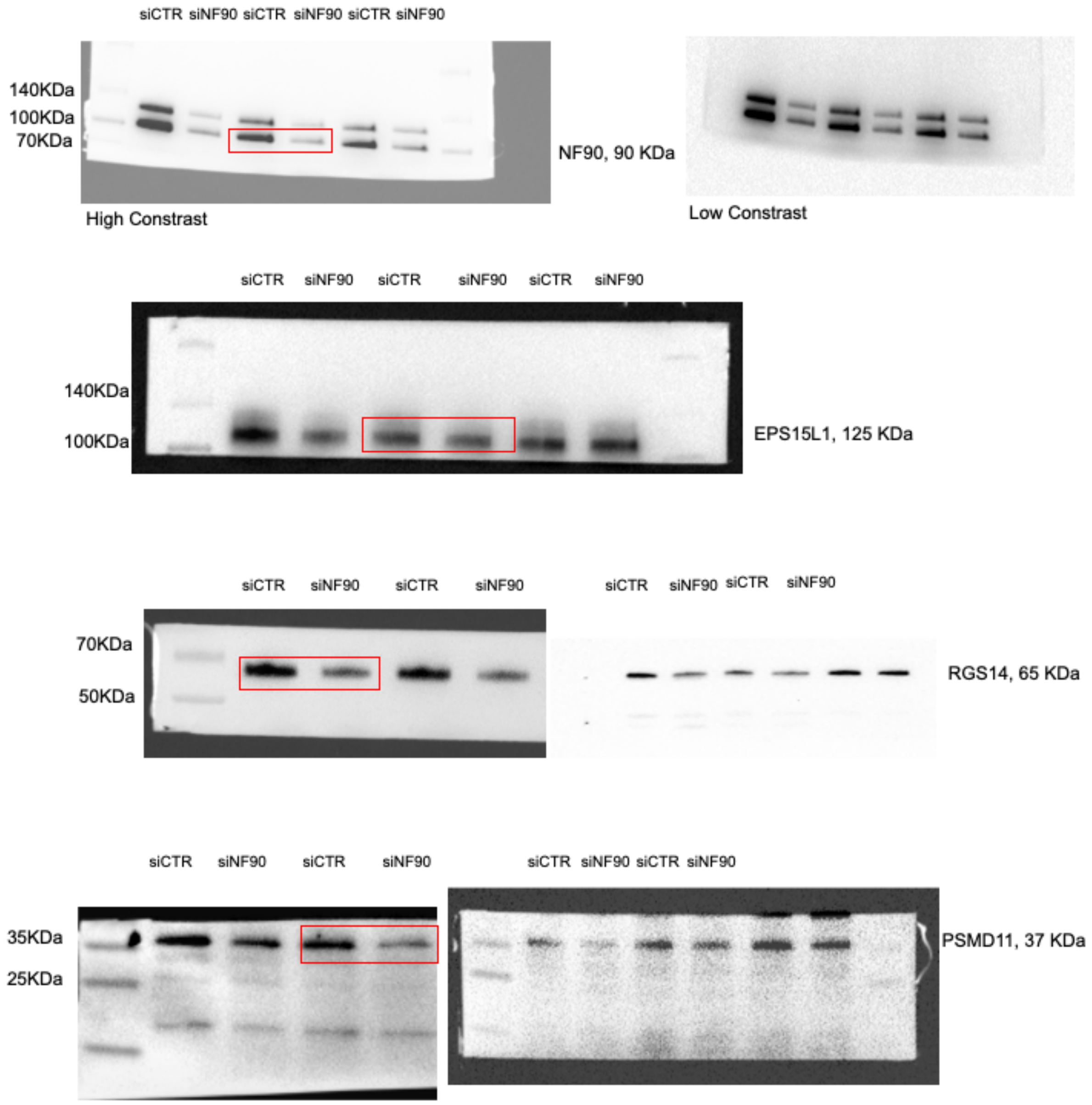

Lodde et al., Fig. 4B

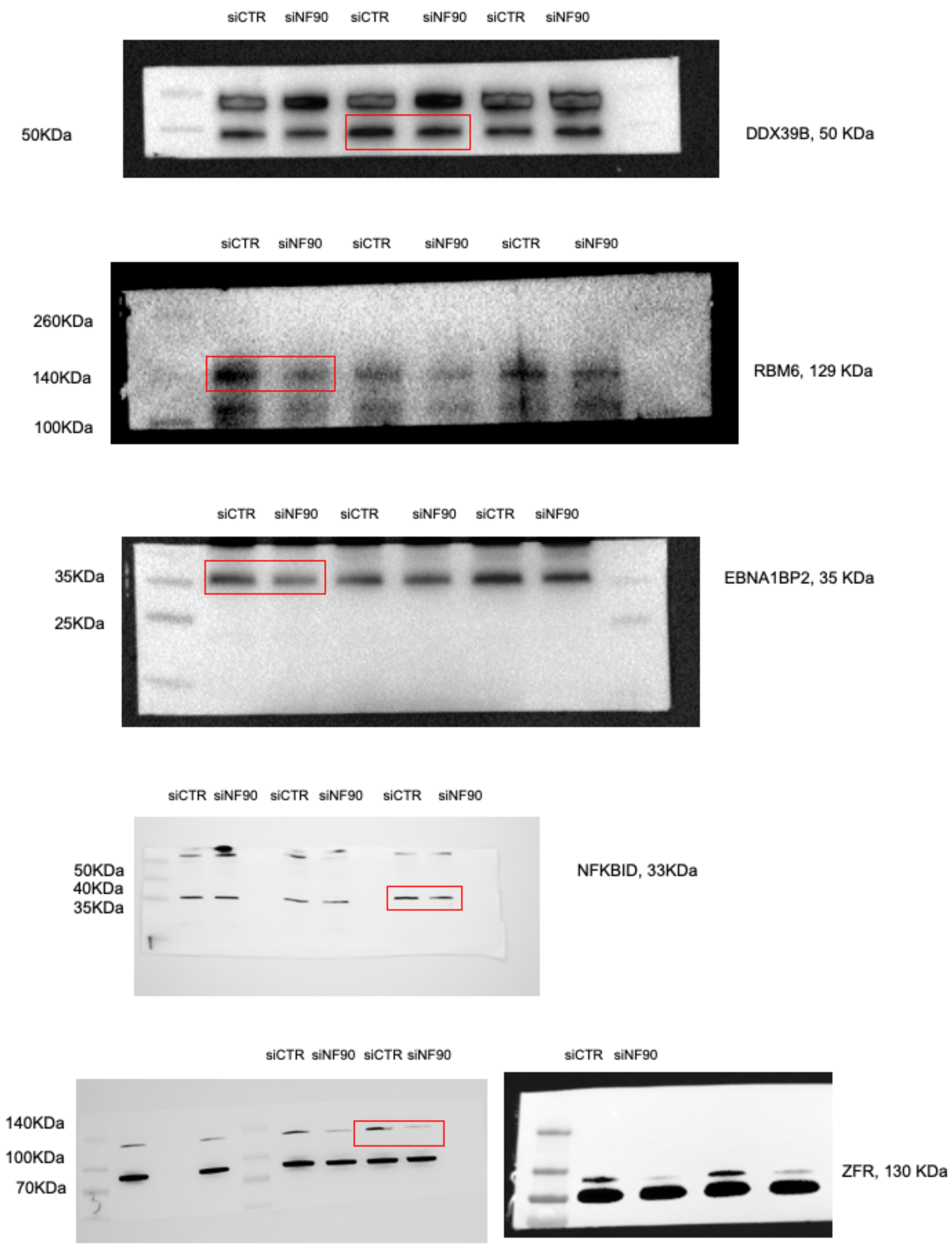

Lodde et al., Fig. 4B

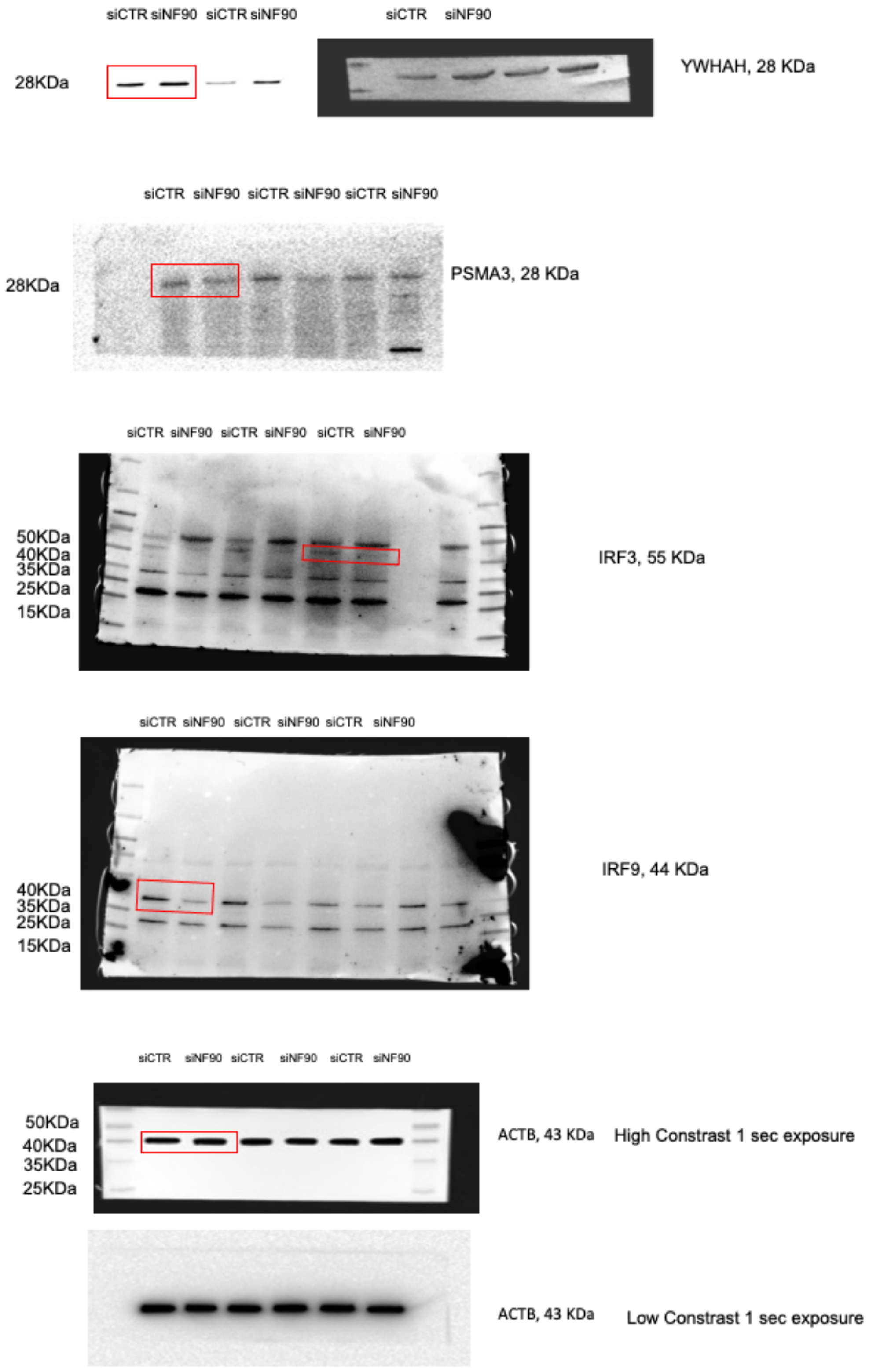

Lodde et al., Fig. 7A

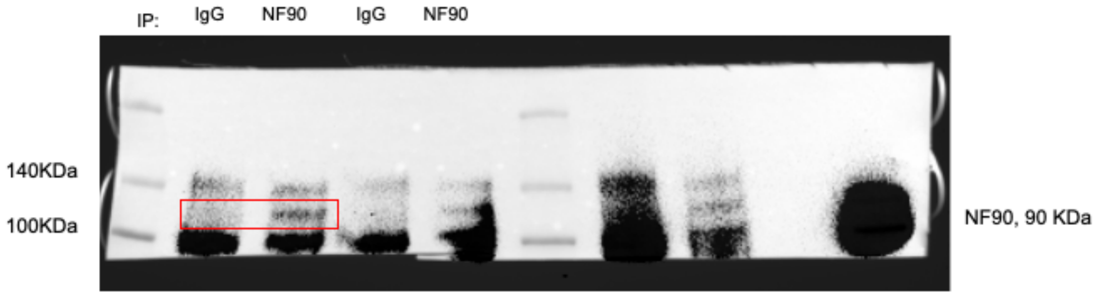

Lodde et al., Fig. 7C

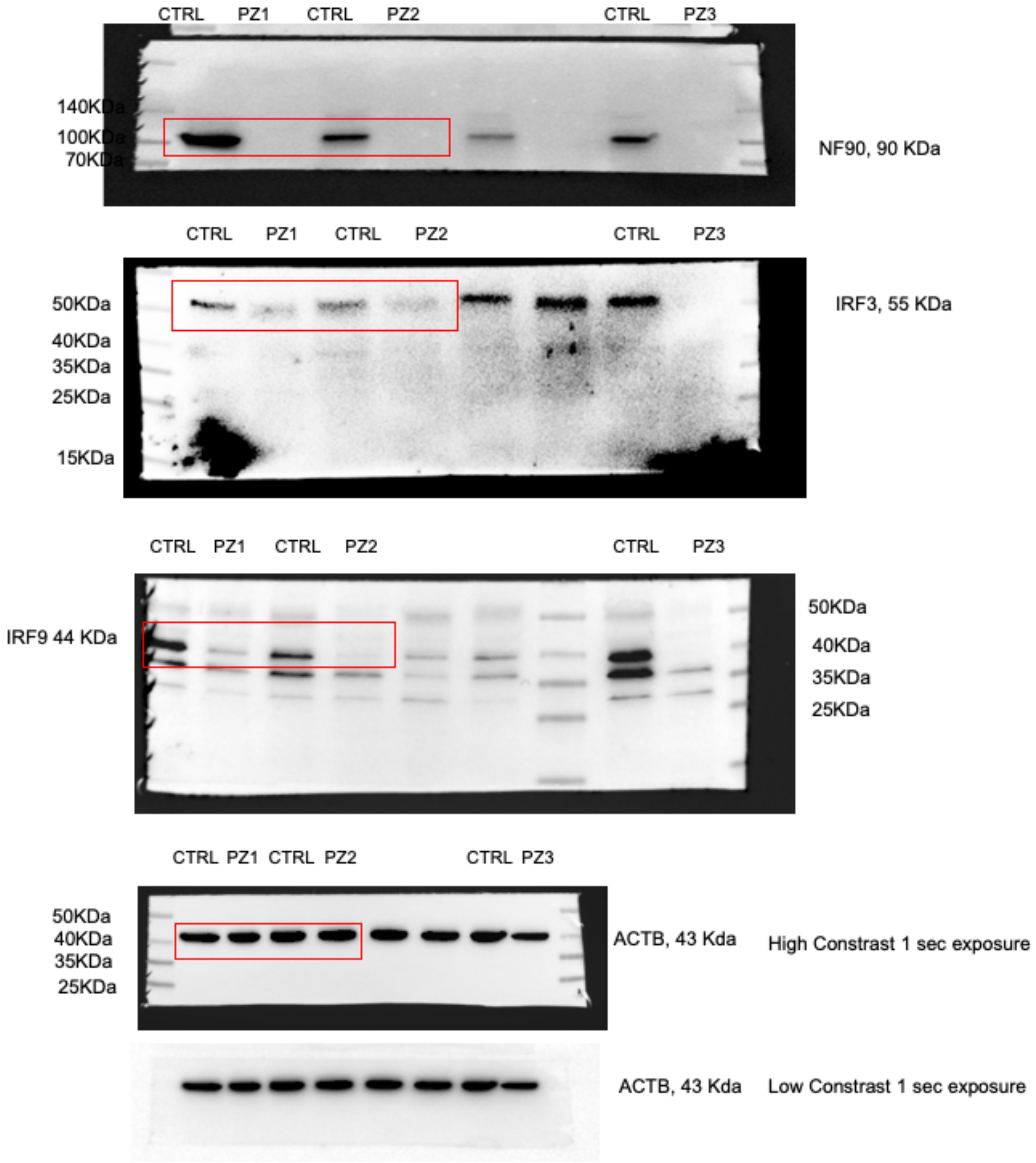

Lodde et al., Supplemental Fig. S3 A

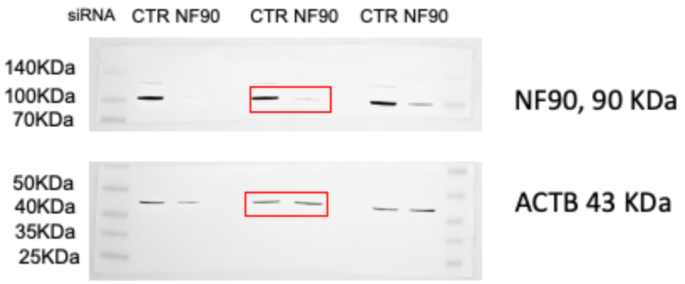

Supplemental Figure legends

Supplemental Figure S1. Characterization of NF90 target mRNAs and binding sites identified by iCLIP in HEK-293 cells. (A) The distribution of NF90 signals throughout all the chromosome (Chr.) was analyzed and plotted as percent of overlapping reads. **(B)** Graphic representation of the coverage profile of query regions at feature boundaries. **(C)** Motif probability matrix (by HOMER) computed from enriched RNAs with NF90 iCLIP-binding sites. Five predominant motifs were identified, two of them are highly A- and U-rich. (**D**) KEGG pathway enrichment analysis of the NF90-target mRNAs identified by iCLIP.

Supplemental Figure S2. Gene ontology (GO) analysis of the targets identified by iCLIP analysis. GO analysis of iCLIP targets identified in our analysis and divided in (A) cellular component and (B) biological process. The diameter of the dots denotes the number of the genes in each subgroup; the colors indicate the p-adjusted value.

Supplemental Figure S3. NF90 silencing modulates the level of target long noncoding (lnc)RNAs. (A) RIP analysis was conducted using lysates from HEK-293 cells and anti-NF90 or IgG antibodies. The levels of enrichment of target lncRNAs were quantified by RT-qPCR analysis after normalization to *ACTB* mRNA. **(B)** In HEK-293 cells expressing normal (Ctrl siRNA) or reduced (NF90 siRNA) NF90 protein levels, the abundance of select lncRNAs was measured by RT-qPCR analysis. (**C**) Genome browser snapshot shows the primary data and signal in a portion of Xist. Data in are the means and standard deviation (+SD) from at least three independent experiments. *, $P < 0.05$; **, $P < 0.01$, ***, $P < 0.005$.

Supplemental Figure S4. RNA-seq analysis of HEK-293 cells expressing different levels of NF90. (A) Western blot analysis of NF90 expression in HEK-293 cells 48 h after transfection of either Ctrl or NF90 siRNAs. **(B)** Volcano plot representation of differentially abundant RNAs in HEK-293 (NF90 siRNAs vs Ctrl siRNA) in the two different comparison groups; reduced (green) and increased (red) mRNAs are highlighted. **(C)** Table indicating the RNAs showing higher and lower abundance after silencing NF90. **(D)** Among the mRNAs showing differential abundance, those displaying the greatest fold increases (red) and decreases (green) by RNA-seq analysis were plotted. **(E)** KEGG pathway enrichment analysis of the mRNAs differentially expressed in HEK-293 cells containing normal or reduced NF90 levels. **(F)** Venn diagram showing the overlap between iCLIP target and transcript differentially expressed by RNA sequencing.

Supplemental Figure S5. Original full-length western blots corresponding to the main figures shown in the manuscript. The cropped area used in the manuscript is shown by a red line frame in the original full western blots.

Supplemental Table legends

Supplemental Table S1. List of peaks from NF90 iCLIP in HEK-293 cells experiment.(cutof: foldchenge > 0.5).

Supplemental Table S2. Gene significativelly upregulate and downregulated identified in RNA sequencing in HEK-293 ctr compared to HEK-293 NF90 siRNA (cutof: foldchenge > 0.5).
